# Targeting C21orf58 is a Novel Treatment Strategy of Hepatocellular Carcinoma by Disrupting the Formation of JAK2/C21orf58/STAT3 Complex

**DOI:** 10.1002/advs.202306623

**Published:** 2024-02-11

**Authors:** Hao Jiang, Yang Wang, Doudou Wen, Rongji Yu, Sayed S Esa, Kefeng Lv, Qing Feng, Jing Liu, Faxiang Li, Lan He, Xiaotang Di, Shubing Zhang

**Affiliations:** ^1^ Department of Biomedical Informatics School of Life Sciences Central South University Changsha Hunan 410013 P. R. China; ^2^ Department of Cell Biology School of Life Sciences Central South University Changsha Hunan 410013 P. R. China; ^3^ School of Biomedical Science Hunan University Changsha Hunan 410013 P. R. China; ^4^ Department of Biochemistry and Molecular Biology School of Life Sciences Central South University Changsha 410013 P. R. China; ^5^ Center for Medical Genetics School of Life Sciences Central South University Changsha 410013 P. R. China

**Keywords:** C21orf58, drug resistance, hepatocellular carcinoma, JAK2/STAT3 pathway, malignant growth, therapy

## Abstract

Hepatocellular carcinoma (HCC) is the third leading cause of cancer‐related death worldwide. Functionally uncharacterized genes are an attractive repository to explore candidate oncogenes. It is demonstrated that C21orf58 displays an oncogenic role in promoting cell growth, tumorigenesis and sorafenib resistance of HCC cells by abnormal activation of STAT3 signaling. Mechanistically, a novel manner to regulate STAT3 signaling that adaptor C21orf58 forms a ternary complex is reveal with N‐terminal domain of STAT3 and SH2 domain of JAK2, by which C21orf58 overactivates wild‐type STAT3 by facilitating its phosphorylation mediated by JAK2, and hyper‐activates of constitutively mutated STAT3 due to preferred binding with C21orf58 and JAK2. Moreover, it is validated that inhibition of C21orf58 with drug alminoprofen, selected by virtual screening, could effectively repress the viability and tumorigenesis of HCC cells. Therefore, it is identified that C21orf58 functions as an oncogenic adaptor, reveal a novel regulatory mechanism of JAK2/STAT3 signaling, explain the cause of abnormal activity of activated mutants of STAT3, and explore the attractive therapeutic potential by targeting C21orf58 in HCC.

## Graphical abstract

Adaptor C21orf58 formed a ternary complex with signal transducer and activator of transcription 3 (STAT3) and Janus kinase 2 (JAK2) to activate wild‐type and constitutively mutated STAT3, enhancing the capacities of malignant growth and sorafenib resistance to hepatocellular carcinoma (HCC) cells. Moreover, targeting C21orf58 showed a significant inhibition on the tumorigenesis of HCC.

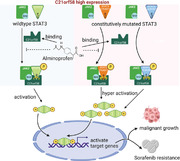

## 1 Introduction

HCC is one of the most common carcinomas worldwide.^[^ ^1^ ^]^ Although effective therapeutic strategies have been developed, the limited efficacy of existing treatments and drug resistance are also the major obstacles to successful treatment of HCC.^[^ ^2^ ^]^ Mounting evidence demonstrates that obvious genetic abnormalities and various disordered signaling pathways are observed in HCC.^[^ ^3^ ^]^ Therefore, identification of the gene involved in carcinogenesis of HCC and clarification of the underlying mechanisms will contribute to understanding the pathogenesis of HCC and developing more effective therapeutic strategies for HCC.

It has been suggested that 5–10% or more of the ≈25 000 putative genes encoded in the human genome probably contribute to oncogenesis.^[^ ^4^ ^]^ However, only a small proportion of them have been experimentally confirmed to causally implicated in cancers.^[^ ^5^ ^]^ C21orf58, expressed by chromosome 21 open reading frame 58 and located in 21q22.3, is a functionally uncharacterized gene.^[^ ^6^ ^]^ The main isoform consists of 216 amino acids, and is mainly located in the cytoplasm, mitochondria, and nucleus of the cell. Serological screening and genome‐wide study indicate that C21orf58 gene is abnormally expressed in lung adenocarcinoma and breast cancer, and related with the malignancy.^[^ ^7^ ^]^ Although these studies imply that C21orf58 may play a vital role in cancer, its function in cancer is completely unknown.

Besides, the analysis results of GEPIA website show that the expression of C21orf58 is not significantly and correlated with prognosis of breast cancer and lung adenocarcinoma, while negatively correlated with the prognosis of HCC patients, which imply that C21orf58 might play an important role in HCC. Therefore, we investigated the biofunction and molecular mechanism of C21orf58 to provide a new biomarker and therapeutic target in HCC diagnosis and therapy.

Signal transducer and activator of transcription 3 (STAT3) is involved in cell proliferation and survival, metastasis, invasion, angiogenesis, and immunosuppression.^[^ ^8^ ^]^ Aberrantly elevated activity of STAT3 occurs in >70% of human cancers and approximately 60% of HCC.^[^ ^9^ ^]^ STAT3 is often found to be constitutively activated and its activation requires phosphorylation of a critical tyrosine residue (Tyr705), which is most commonly mediated by Janus kinases (JAKs), especially JAK2.^[^ ^10^ ^]^ Many studies indicate that sustained activation of STAT3 could promote HCC development, and STAT3‐positive HCC is more aggressive and drug‐resistant.^[^ ^11^ ^]^ Thus, STAT3 becomes an attractive molecule target, but the overall anti‐tumor effects of a number of different STAT3 inhibitors have not been overly impressive.^[^ ^12^ ^]^ Moreover, although the crucial roles of STAT3 in tumor cells and microenvironment are evident,^[^ ^13^ ^]^ gaps between upstream regulators and STAT3 remain exist. Therefore, exploring novel regulation mechanism and novel inhibitors of STAT3 signaling are helpful for the treatment and prevention of human malignancies.

Many uncharacteristic genes may be involved in the occurrence and progression of tumors. The characteristics of C21orf58 are still unclear in cancers. Our study explored the biological function, underlying mechanism, and therapeutic potential of C21orf58 in HCC. In this study, we clarified the oncogenic biofunction of C21orf58, illuminated the underlying molecular mechanism of prompting growth, tumorigenesis, and sorafenib resistance mediated by C21orf58 in HCC cells, identified a new adaptor and a novel regulation mechanism of STAT3 signaling, explained the cause of hyper‐activation of STAT3 driven by constitutive mutations, and evaluated the therapeutic potential of targeting C21orf58 in HCC.

## 2 Results

### 2.1 C21orf58 Expression Was Upregulated in HCC Tissues and Positively Correlated with Poor Prognosis

**Figure 1:**
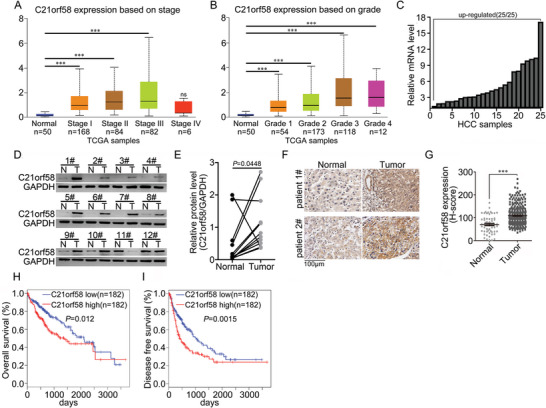
High expression of C21orf58 was observed in HCC tissues and predicted poor prognosis of HCC patients. A,B) C21orf58 expression in HCC samples with different stages and grades was analyzed using UALCAN database. C) C21orf58 mRNA level was examined by qPCR in 25 pairs of HCC tissues and matched non‐carcinoma hepatic tissues. D,E) C21orf58 protein level in pairs of HCC tissues and matched non‐carcinoma hepatic tissues. The intensity of C21orf58 protein was analyzed by ImageJ software. F) Immunohistochemistry staining of C21orf58 in normal hepatic tissues and HCC tissues. Scale bar, 100 µm. G) The H‐score value of C21orf58 staining intensity in normal hepatic tissues (*n* = 60) and HCC tissues (*n* = 320). The data were analyzed using Student's *t*‐test. H,I) The Overall survival and disease‐free survival of HCC patients was analyzed by using GEPIA database. Survival curves were plotted by the Kaplan‐Meier method and analyzed by the log‐rank test. All *** *P*<0.001.

Both exome‐wide association study and genome‐wide DNA methylation analysis hinted the involvement of C21orf58 in cancer.^[^ ^7^, ^14^ ^]^ Based on the analysis results of ULCAN database, we found that C21orf58 expression was upregulated in HCC with higher pathological grade and tumor stage (**Figure** **1**A,B), which implied that C21orf58 expression was abnormal and associated with HCC progression. To confirm our hypothesis, C21orf58 mRNA and protein levels in paired HCC tissues were examined, and both of them were highly expressed in HCC tissues compared with matched adjacent hepatic tissues (Figure 1C–E). Consistently, higher expression of C21orf58 protein was detected in most of the HCC tissues compared to adjacent non‐tumor tissues (*P* < 0.001) (Figure 1F,G). According to C21orf58 expression levels, HCC patients were divided into C21orf58 high (H‐score≥50) and low (H‐score<50) expression groups, and the correlation between C21orf58 expression and clinical pathology features was assessed. As shown in **Table** **1**, C21orf58 expression was positively correlated with tumor size (*P* = 0.042). Moreover, clinical prognostic analysis indicated that patients with high C21orf58 expression were predicted with worse overall survival (*P* = 0.023) (Figure S1A, Supporting Information) and disease‐free survival (*P* = 0.0159) (Figure S1B, Supporting Information). GEPIA data further confirmed that C21orf58 expression was positively associated with poor prognosis of HCC patients (*P* = 0.012, *P* = 0.0015) (Figure 1H,I). Taken together, these results suggested that C21orf58 was significantly upregulated in HCC tissues and exerted an oncogene role on prognosis of patients.

**Table 1:** Correlation between the C21orf58 expression and the clinicopathologic features of hepatocellular carcinoma (HCC) (Analysis was performed based on 320 cases of HCC using Chi‐square χ2 test. Staining intensity (> = 50) was used as the cut off point for the high expression and low expression of C21orf58).

Characteristic	Total	C21orf58(*n*%)		*χ* ^2^	*P* value
		High	Low
	320	*n* = 199	*n* = 121		
Gender				3.738	0.065
Male	285	172	113		
Female	35	27	8		
Age(years)				0.033	0.907
> = 50	129	81	48		
<50	191	118	73		
Tumor size				4.335	0.042^a)^
> = 6	268	160	108		
<6	52	39	13		
Histological differentiation				2.938	0.092
Well	287	183	104		
Poor	33	16	17		
Lymph node metastasis				0.043	0.817
Yes	20	12	8		
No	300	187	113		
TNM stage				0.253	0.608
I‐II	304	190	114		
III‐V	16	9	7		
Tumor number					
> = 2	32	19	13	0.120	0.848
< = 1	288	180	108		
Organ metastasis				0.799	0.427
Yes	81	47	34		
No	239	152	87		

^a)^ *P* < 0.05.

### 2.2 C21orf58 Promoted the Growth and Tumorigenesis of HCC Cells

**Figure 2:**
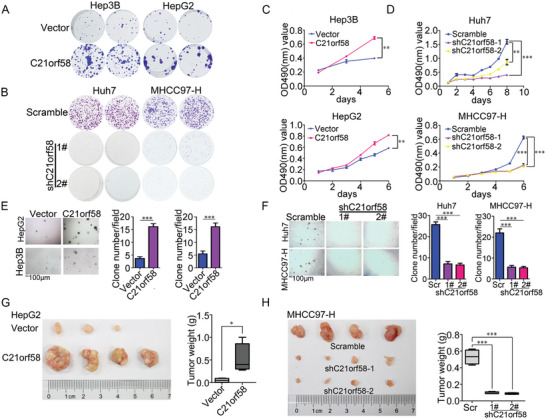
C21orf58 enhanced the growth and tumorigenesis of HCC cells. (A–D) The growth‐promoting effect of C21orf58 in HCC cells was examined by crystal violet and MTT assays. E,F) The effect of C21orf58 on clone formation of HCC cells was investigated by soft agar assay. Scale bar, 100 µm. G) Overexpression of C21orf58 increased the volume and weight of tumors formed by HepG2 cells. H) Knockdown of C21orf58 reduced the volume and weight of MHCC97‐H derived tumors. All * *P*<0.05, ** *P*<0.01, *** *P*<0.001.

The clinical data implied that C21orf58 might be associated with the growth ability of HCC. Therefore, we established the stable HCC cell lines, which were overexpressed or knocked down C21orf58 (Figure S2A,B, Supporting Information). As revealed by crystal violet and MTT assays, we observed that overexpression of C21orf58 significantly enhanced the growth of HCC cells (**Figure** **2**A,C), while knockdown of C21orf58 remarkably inhibited HCC cell growth (Figure 2B,D). Moreover, forced expression of C21orf58 significantly promoted the colony formation of HCC cells (Figure 2E). In contrast, downregulation of C21orf58 expression obviously suppressed the ability of colony formation (Figure 2F). Additionally, cell‐derived xenograft experiment indicated that C21orf58 overexpression notably increased the volume and weight of HCC cells formed tumors (Figure 2G), attenuated C21orf58 notably decreased the tumor size and weight (Figure 2H), which were consistent with the results in vitro. Taken together, our data were consistent with the increased expression of C21orf58 in HCC tissues and demonstrated that C21orf58 displayed a vital role on malignant growth and tumorigenesis of HCC cells.

### 2.3 C21orf58 Obviously Increased the Expression Level of Phosphorylation of STAT3

**Figure 3:**
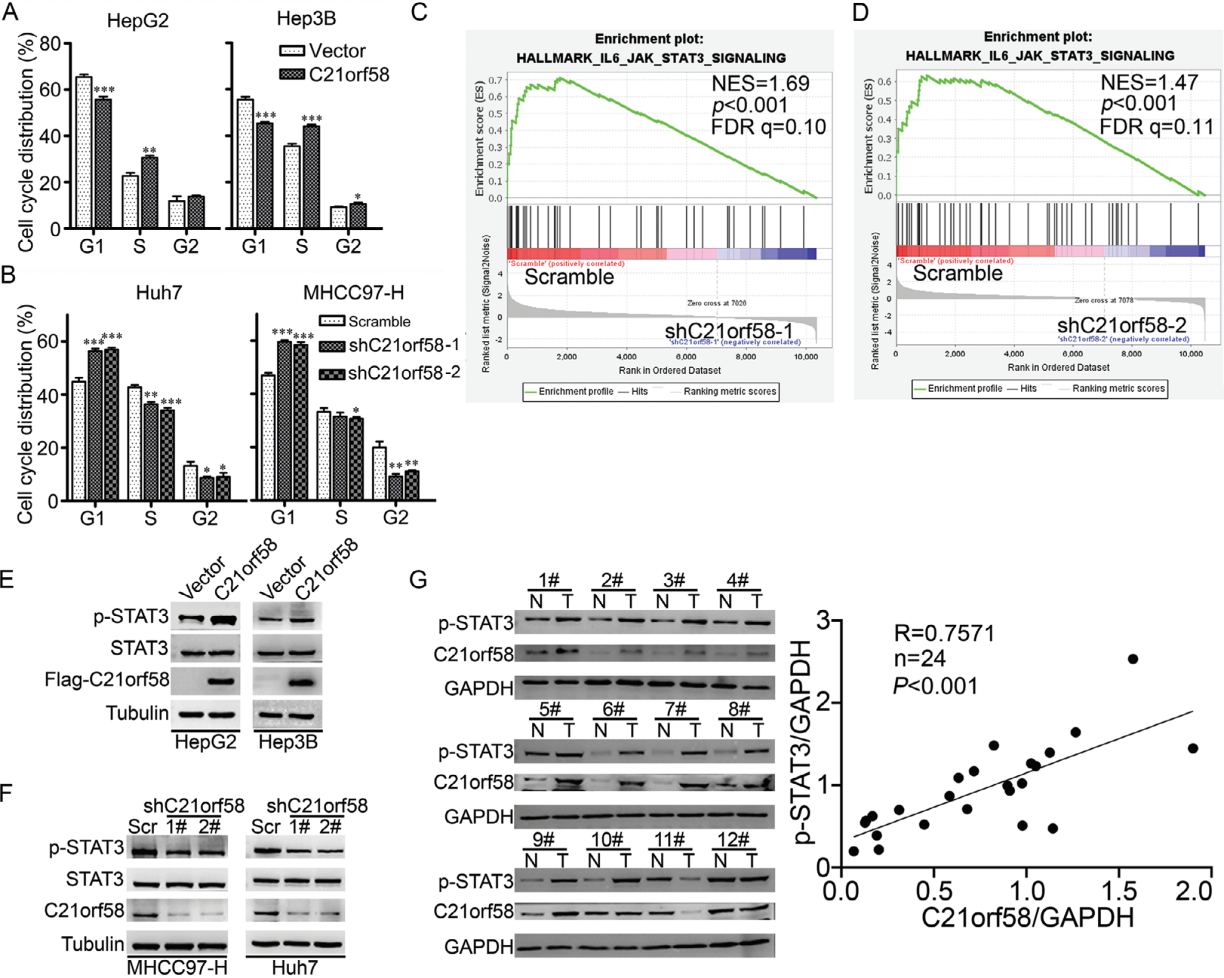
C21orf58 accelerated cell cycle of HCC cells and increased the expression of phosphorylated STAT3. A,B) The effects of C21orf58 overexpression and knockdown on cell cycle distribution of HCC cells. C,D) The gene set enrichment analysis (GSEA) plot of IL6‐JAK‐STAT3 signaling pathway based on the RNA seq data from control and C21orf58 knockdown HCC cells (shC21orf58‐1 and shC21orf58‐2, *n* = 3 per group). NES, normalized enrichment score. E,F) STAT3 and p‐STAT3(Y705) protein levels in C21orf58 overexpression and knockdown HCC cells. G) The expression levels of C21orf58 and p‐STAT3(Y705) proteins in paired clinical HCC tissues (*n* = 12). The positive correlation between C21orf58 and p‐STAT3(Y705) expression was assessed by linear regression. All * *P*<0.05, ** *P*<0.01, *** *P*<0.001. Scr: Scramble.

To investigate the function of C21orf58 on cell growth, we examined the cell cycle of stable cells. Upregulated C21orf58 expression remarkably decreased the percentage of G1‐phase cells, and raised the percentage of S‐phase cells (**Figures** **3**A and S3A, Supporting Information). Meanwhile, reverse results were observed in C21orf58 knockdown cells (Figure 3B and Figure S3B, Supporting Information). These results indicated that C21orf58 accelerated G1/S phase transition of HCC cell cycle. Then, we performed RNA‐sequence using C21orf58 knockdown and control cells, GSEA, KEGG and GO enrichment assays hinted that C21orf58 expression was positively correlated with IL‐6/JAK2/STAT3 and JAK/STAT signaling (Figure 3C,D and Figure S4A–C, Supporting Information). In fact, when C21orf58 was overexpressed, the expression of p‐STAT3 and p‐JAK2 proteins were remarkably increased (Figure 3E and Figure S3C, Supporting Information), and the transcriptions of STAT3 targeted genes (such as IL6 and CyclinD1) were elevated (Figure S3E, Supporting Information), but total STAT3 and JAK2 as well as EGFR pathway were not affected (Figure S3D, Supporting Information). Conversely, both p‐STAT3 and p‐JAK2 were downregulated when C21orf58 expression was knocked down (Figure 3F and Figure S3C, Supporting Information). In addition, the co‐expression correlation between C21orf58 and p‐STAT3 was significant in clinical HCC tissues (*P* < 0.001) (Figure 3G). Our results identified that C21orf58 promoted cell cycle by accelerating G1/S phase transition and enhanced the expression of p‐STAT3, which suggested that C21orf58 might endow HCC cells with malignant growth ability by modulating the activity of STAT3 signaling.

### 2.4 Adaptor C21orf58 Simultaneously Interacted with N‐Terminal Domain of STAT3 and SH2 Domain of JAK2 to Form a Ternary Complex

**Figure 4:**
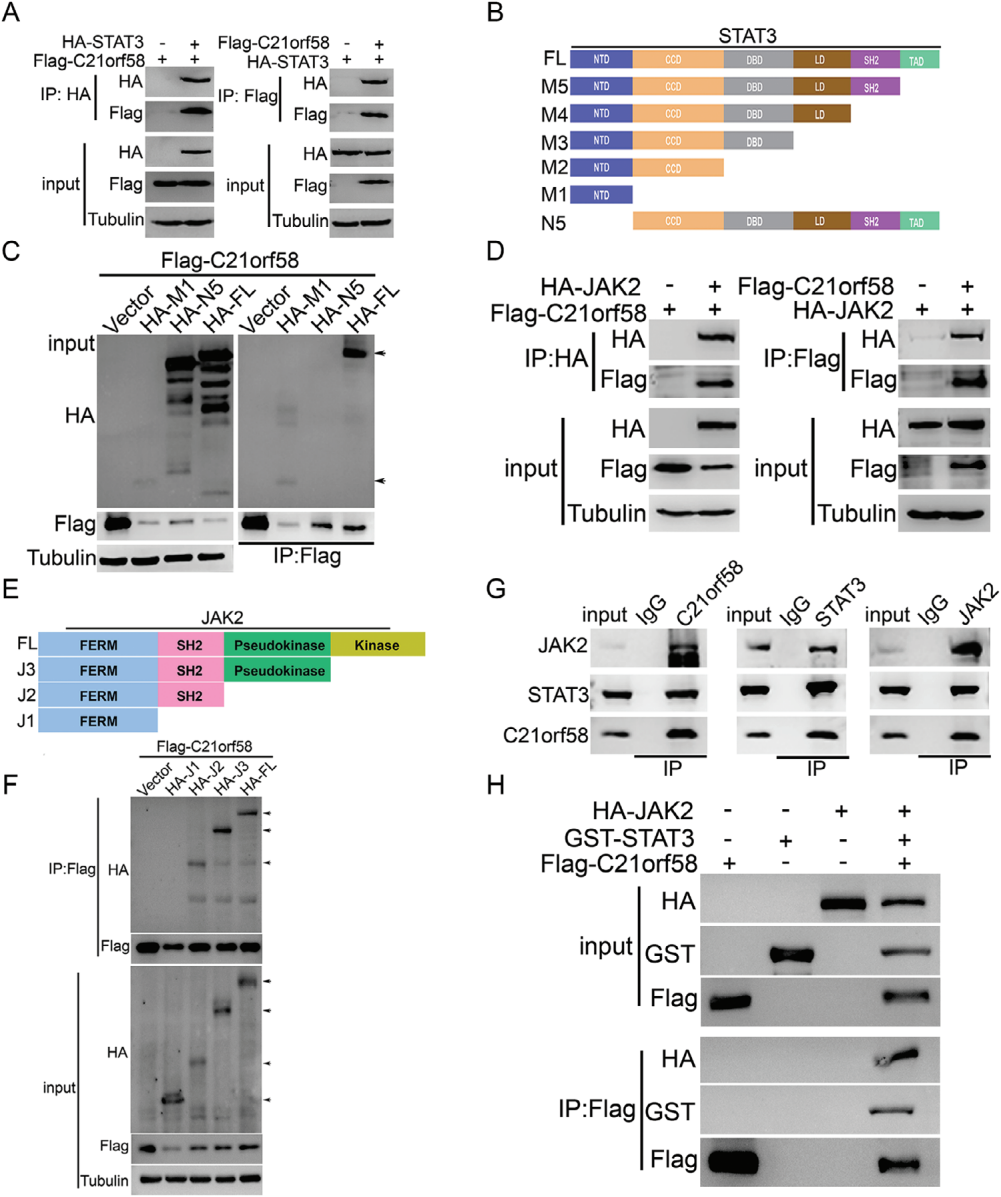
C21orf58 simultaneously interacted with JAK2 and STAT3 to form a ternary complex. A) The exogenous interaction between C21orf58 and STAT3 in HepG2 cells. B) Truncations of STAT3 were constructed as shown in graphic, NTD: N‐terminal domain; CCD: coiled‐coil domain; DBD: DNA‐binding domain; LD: linker domain; SH2: SH2 domain; TAD: transactivation domain. C) The interaction between C21orf58 and NTD domain of STAT3 was validated by immunoprecipitation. D) The exogenous interaction between C21orf58 and JAK2 in HepG2 cells. E) Truncations of JAK2 were constructed as shown in graphic. F) Interaction domains between JAK2 and C21orf58 was detected by immunoprecipitation, SH2 domain was the binding region of C21orf58 on JAK2. G) Co‐immunoprecipitation showed that C21orf58 simultaneously interacted with JAK2 and STAT3 in HepG2 cells. H) In vitro pull‐down assay was performed to conform that C21orf58 formed a ternary complex with JAK2 and STAT3 in HCC cells by direct interaction.

To explore how C21orf58 regulated the phosphorylation of STAT3, we performed the co‐immunoprecipitation (Co‐IP) and revealed the interaction proteins of C21orf58 by mass spectrometry assay. Notably, JAK2 and STAT3 were detected among the candidate binding proteins (Figure S5A, Supporting Information). Then, Co‐IP assay was used to validate the binding between C21orf58 and STAT3, and results showed that C21orf58 interacted with STAT3 either exogenously or endogenously (**Figure** **4**A and Figure S5B, Supporting Information), which was further confirmed by their colocalization in the HCC cells (Figure S5C, Supporting Information). Moreover, various truncated mutants of STAT3 were constructed (Figure 4B), and truncation assay demonstrated that N‐terminal domain (NTD) of STAT3 was indispensable for C21orf58‐STAT3 interaction (Figure S5D, Supporting Information and Figure 4C). Similarly, the binding between C21orf58 and JAK2 was assessed. We also found that C21orf58 mutually interacted with JAK2 (Figure 4D), and truncation assay further revealed that C21orf58 bound to the SH2 domain of JAK2 (Figure 4E,F). It was reported that the root‐mean‐square deviation (RMSD) of molecular dynamics simulation could provide insight into the motion process of a complex. The RMSD plots of C21orf58, STAT3, and JAK2 were generated and the RMSD values of C21orf58 fluctuated from 0 ns to 10 ns, then showed stability after 10 ns, which declined from 3 to 2.2 Å after 10 ns. Furthermore, STAT3 and JAK2 showed slight fluctuation at 20 ns, then showed stability, from which C21orf58, STAT3 and JAK2 showed strong stability (Figure S6A, Supporting Information). To predict the local fluctuations of macromolecular proteins at the residue level, root mean square fluctuation (RMSF) was computed. The graph presented in Figure S6B (Supporting Information) illustrates the extent of fluctuation in RMSF for each residue within the proteins C21orf58, STAT3, and JAK2. Residues situated in the loop regions, specifically those found at the N‐ and C‐terminals, demonstrated more pronounced fluctuations in comparison to other areas. On the other hand, the alpha helices and beta strands exhibited heightened structural stability, resulting in lesser fluctuations across all three protein complexes. The observed RMSF values, ranging from 1.0 to 7.0 Å, provided valuable insights into the robustness and steadiness of these protein complexes (Figure S6B, Supporting Information). Elevated numbers of hydrogen bonds were detected in Figure S6C (Supporting Information), conferring stability to the protein structure and providing the specificity required for selective macromolecular interactions, which led to greater stability of protein–protein interactions. The persistent presence of at least two hydrogen bonds throughout the simulation strongly suggested robust and enduring binding stability between C21orf58 and STAT3, JAK2 and C21orf58, as well as JAK2 and STAT3 (Figure S6C, Supporting Information). Obtained results implied that these three proteins composed a complex, and C21orf58 might interact with the JAK2 and STAT3 simultaneously. To verify it, endogenous Co‐IP was performed and detected that JAK2, STAT3 and C21orf58 could interact with each other (Figure 4G, Supporting Information). Moreover, IP‐re‐IP assay determined that C21orf58 simultaneously bound with JAK2 and STAT3, forming a novel complex (Figure S5E, Supporting Information). Fundamentally, in vitro protein pull‐down assay further confirmed the direct interaction of C21orf58, STAT3 and JAK2, and they formed the ternary complex (Figure 4H). In general, our results clarified that C21orf58 displayed an adaptor role to make JAK2 and STAT3 form a ternary complex by directly interacting with NTD and SH2 domain.

### 2.5 C21orf58 Displayed an Oncogenic Role on Facilitating Overactivation of Wildtype and Constitutively Mutated STAT3 by Promoting the Formation of Ternary Complex

**Figure 5:**
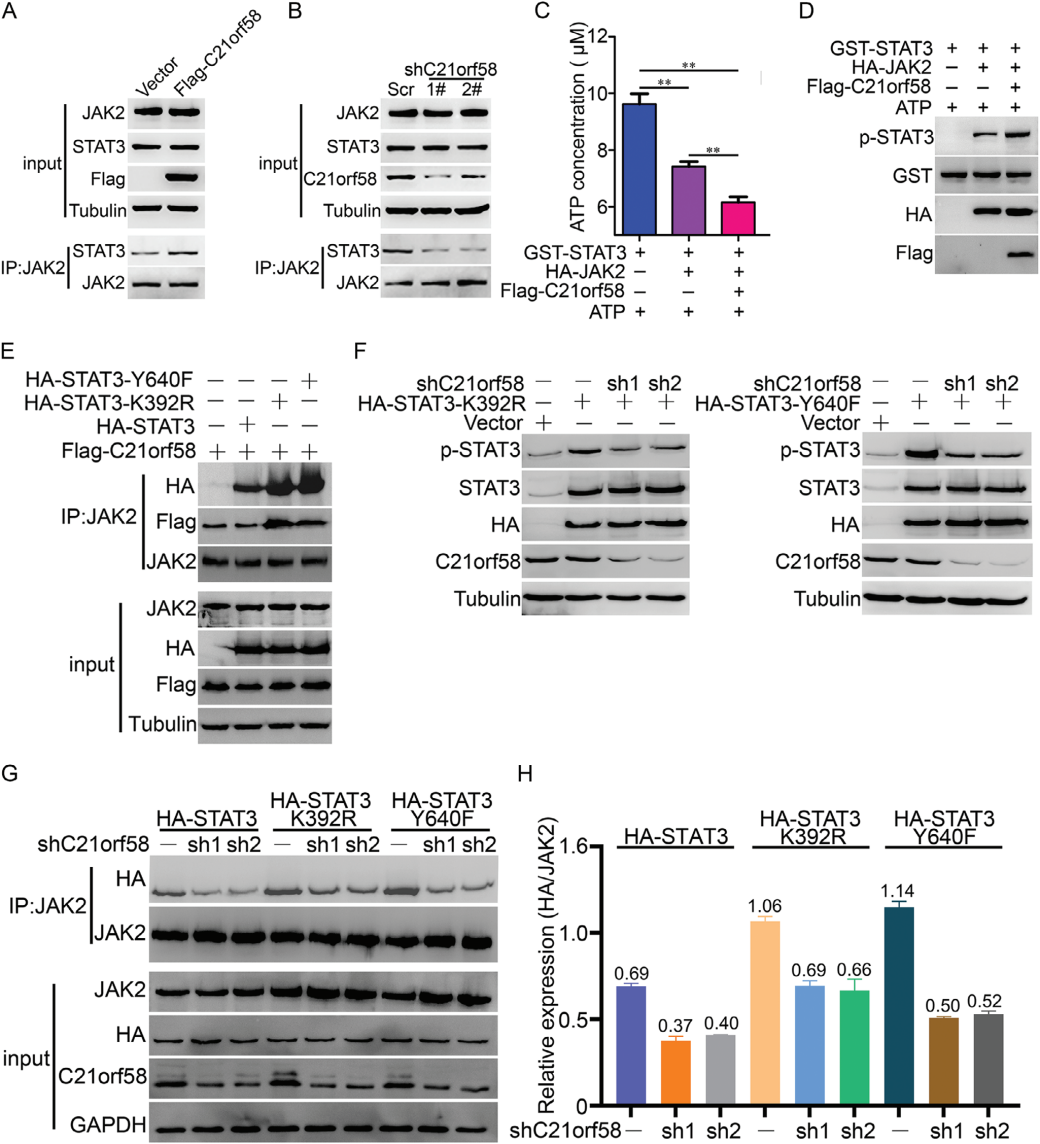
C21orf58 facilitated the activity of wildtype and constitutively mutated STAT3 by forming ternary complex. A) C21orf58 overexpression promoted the interaction of JAK2 on STAT3. B) Attenuated C21orf58 expression decreased the interaction of JAK2 on STAT3. C) In vitro kinase activity assay was performed to verify that C21orf58 promoted the phosphorylation of STAT3 by JAK2. D) After kinase activity assay, proteins were examined by western blot and detected that C21orf58 improved the phosphorylation of STAT3 by JAK2. E) C21orf58 improved the interaction between JAK2 and constitutively activated mutants of STAT3. F) Reduction of C21orf58 expression remarkably declined the phosphorylation of constitutively mutated STAT3. G,H) Downregulation of C21orf58 effectively reduced the interaction of JAK2 on constitutively activated mutants of STAT3.

Next, we investigated whether C21orf58 unduly increased the activity of STAT3 by formation of ternary complex, which made JAK2 easily phosphorylate STAT3. It was observed that the interaction between JAK2 and STAT3 was notably elevated when C21orf58 was overexpressed (**Figure** **5**A), while was significantly reduced when C21orf58 was attenuated (Figure 5B). Meanwhile, in vitro kinase activity assay showed that the consumption of ATP was obviously elevated when C21orf58 appeared (Figure 5C), and western blot indicated that phosphorylation of STAT3 mediated JAK2 was remarkably upregulated by C21orf58 (Figure 5D). Based on these results, we proved that C21orf58 extraordinarily improved STAT3 activity by enhancing the JAK2‐STAT3 interaction through forming ternary complex, resulting in endowing HCC cells with abnormal growth ability. It was reported that continuously activated STAT3 frequently offered malignant capacities to cancers.^[^ ^15^ ^]^ Therefore, we constructed K392R and Y640F constitutively active mutants of STAT3 (Figure S7A, Supporting Information), and examined the effect of C21orf58 on activity of constitutively‐activated STAT3. Co‐IP assay found that K392R and Y640F STAT3 mutants were more preferable to interact with C21orf58 (Figure S7B, Supporting Information), and C21orf58 fundamentally improved the formation of JAK2/C21orf58/mutated‐STAT3 ternary complex when compared with wildtype STAT3 (Figure 5E). Moreover, knockdown of C21orf58 expression significantly inhibited the activity of constitutively‐activated STAT3 (Figure 5F), because the interactions of JAK2 and STAT3 mutants were distinctly reduced (Figure 5G,H). Our results not only demonstrated that C21orf58 displayed a vital regulator role on modulating STAT3 signaling by forming ternary‐activating complex, but also revealed a novel regulation manner of JAK2/STAT3 signaling.

### 2.6 Activation of STAT3 Signaling Was Responsible for Growth‐Promoting Phenotype Mediated by C21orf58

To further validate that C21orf58 offered the growth‐promoting capacity on HCC cells by activating STAT3 signaling, we performed rescue experiments in stable cells. Knockdown of JAK2 or STAT3 expression effectively suppressed the viability of HCC cells overexpressing C21orf58, reversely, overexpression of JAK2 or STAT3 evidently elevated the growth capacity of HCC cells knocking down C21orf58 expression (Figure S8A,B, Supporting Information). Meanwhile, inhibition of JAK2 or STAT3 respectively using Fedratinib and Stattic also notably repressed the growing activity of HCC cells mediated by C21orf58 (Figure S8C, Supporting Information). Moreover, crystal violet assay confirmed the above results that the colony formation ability of C21orf58‐overexpressed HCC cells was blocked when JAK2 or STAT3 expression was attenuated or their activities were inhibited (Figure S8D,F, Supporting Information). Instead, colony formation ability of C21orf58 knockdown HCC cells was recovered when JAK2 or STAT3 was overexpressed (Figure S8E, Supporting Information). Additionally, the expression pattern of p‐STAT3 and p‐JAK2 proteins further verified that inhibition of JAK2 or STAT3 could rescue the growth phenotype mediated by C21orf58 in HCC (Figure S8G–J, Supporting Information). In general, our results corroborated that C21orf58 promoted the malignant growth of HCC cells via activation of STAT3 signaling.

### 2.7 C21orf58 Promoted the Sorafenib Resistance of HCC Cells

**Figure 6:**
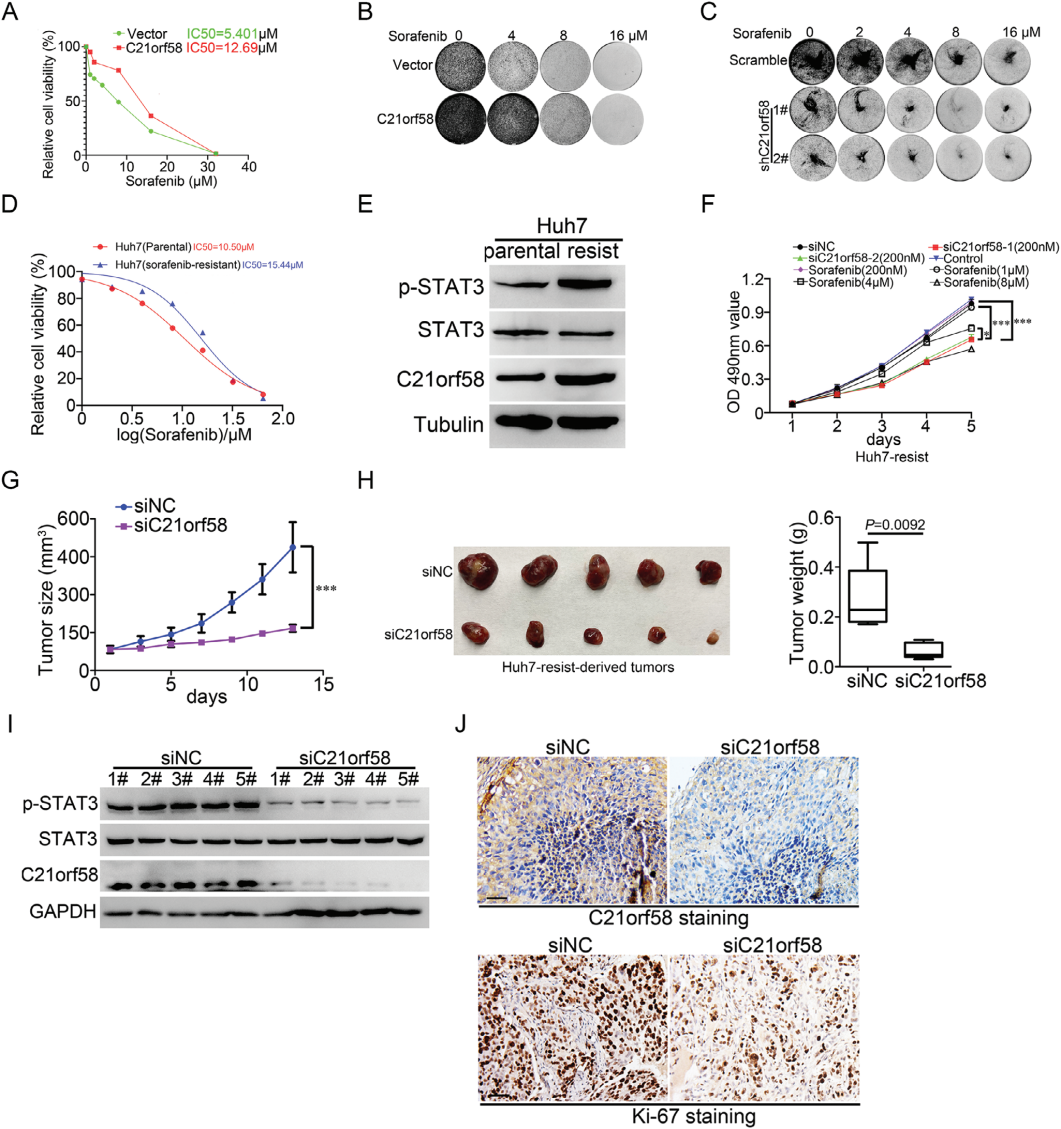
C21orf58 promoted sorafenib resistance of HCC cells. A) C21orf58 elevated the IC50 value of HepG2 cells. B,C) The clone formation of C21orf58 overexpressed and knockdown HCC cells treated with sorafenib at different concentrations. D) Construction of sorafenib‐resistant Huh7 cells, which were not vulnerable to sorafenib compared with their parental cells. IC50 was the 50% inhibiting concentration. E) The expression of C21orf58 and p‐STAT3(Y705) were increased in sorafenib‐resistant and Huh7 cells. F) Inhibition of C21orf58 expression using siRNA was effectively to repress the cell growth of HCC cells with sorafenib resistance. G,H) The growth curve, volume and weight of tumors derived from sorafenib‐resistant Huh7 cells were suppressed by siC21orf58, tumors treated with siC21orf58 (5 nmol) twice a week. I) After treating with siC21orf58 and negative control siRNA respectively, the expression of p‐STAT3(Y705), STAT3 and C21orf58 proteins in sorafenib‐resistant Huh7‐derived tumors were detected by western blot. siRNA processing condition: tumors were treated with siC21orf58 (5 nmol) or negative control siRNA twice a week. J) Immunohistochemistry was performed to investigate the expression of C21orf58 and Ki‐67 proteins. Scale bar = 100 µm. All *** *P*<0.001. siNC: negative control siRNA.

Previous study indicated that STAT3 activation resulted in sorafenib resistance of clinical HCC, therefore, we investigated the association between C21orf58 expression and sorafenib resistance in HCC cells. Compared with control cells, HCC cells overexpressing C21orf58 were less susceptible (**Figure** **6**A) and showed stronger ability of clone formation when treated with sorafenib (Figure 6B), on the contrary, HCC cells knockdown C21orf58 expression were more sensitive to sorafenib (Figure 6C). Moreover, in sorafenib‐resistant HepG2 and Huh7 cells (Figure S9A, Supporting Information and Figure 6D), C21orf58 expression was remarkably upregulated, concurrent with STAT3 activation (Figure 6E and Figure S9B, Supporting Information). These results suggested that C21orf58 mediated the drug resistance and was a potential target of sorafenib‐tolerant HCC cells. Then, we designed two pairs of siRNAs targeting C21orf58 (Figure S9C, Supporting Information), which not only dramatically suppressed the viability of HCC cells with or without sorafenib resistance in vitro (Figure 6F and Figure S9D,E, Supporting Information), but also substantially inhibited the growth capability and reduced the volume and weight of tumors derived from sorafenib‐resistant Huh7 cells (Figure 6G,H). Besides, siC21orf58 obviously prevented the activation of STAT3 (Figure 6I) and reduced the protein level of Ki‐67, which could reflect the rapid proliferation ability of tumors (Figure 6J). In brief, we proved that C21orf58 made HCC cells resist to sorafenib and targeting it could effectively block the malignant growth activity of sorafenib‐tolerated HCC cells.

### 2.8 Alminoprofen, a Ligand of C21orf58, Displayed a Promising Potential in HCC Therapy

**Figure 7:**
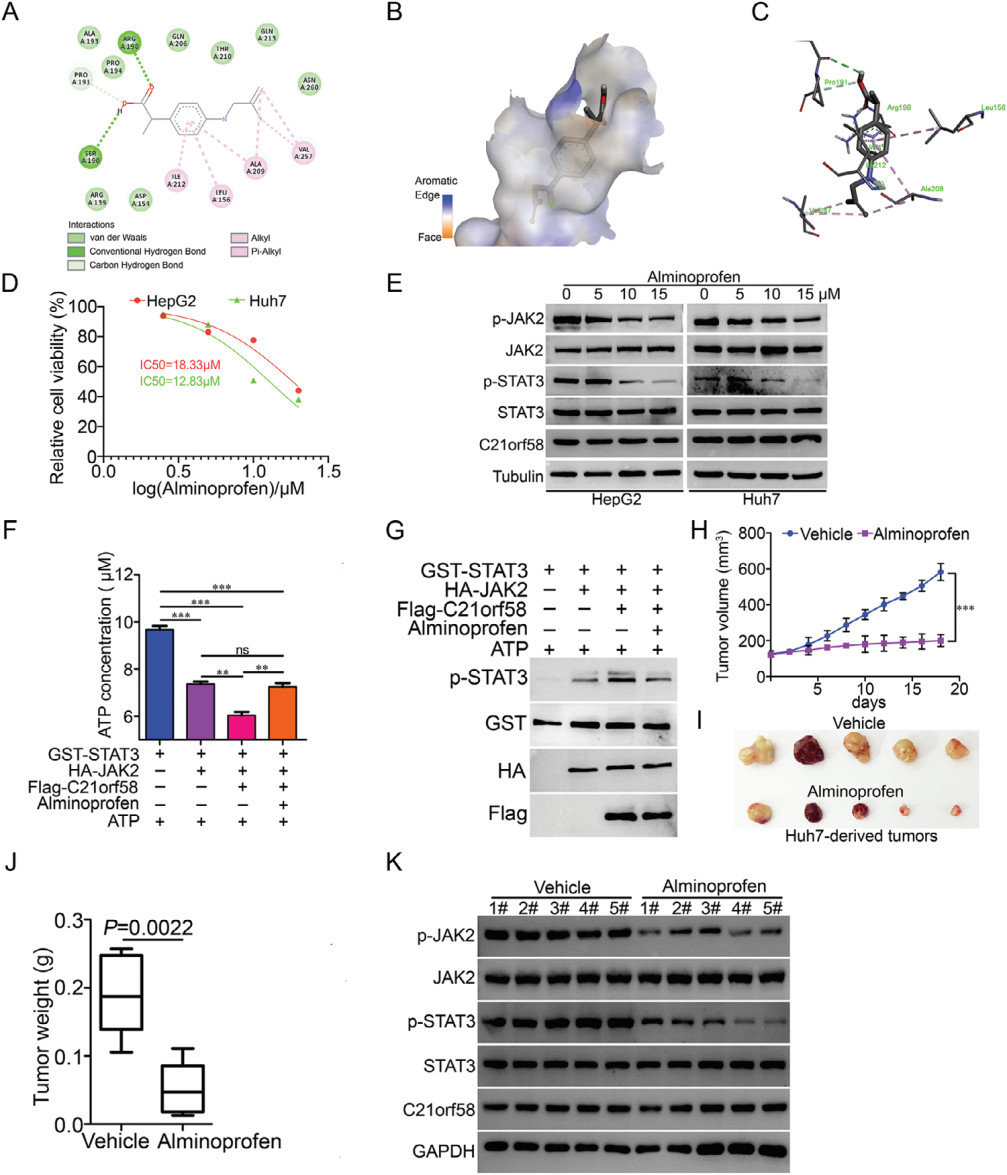
Alminoprofen, a ligand of C21orf58, displayed a promising potential in HCC therapy. A) A 2D hydrogen bond (green dash line) bound the alminoprofen to the amino acid residues of C21orf58. B) 3D model of C21orf58's optimal binding mechanism in the protein pocket (alminoprofen depicted as colored sticks). C) Amino acid residues of C21orf58 interacting with the alminoprofen in 3D (color sticks). D) The inhibitory effect of alminoprofen on cell viability of HepG2 and Huh7 cells by CCK8 assay. E) The effect of alminoprofen on expression of p‐STAT3 and p‐JAK2 proteins in HepG2 and Huh7 cells was examined by western blot. F) Alminoprofen showed a block on ATP consumption mediated by C21orf58 via kinase activity assay in vitro. G) After kinase reaction, the level of p‐STAT3 was investigated by western blot. H) The growth curve of Huh7‐derived tumors treated with alminoprofen (50 mg kg^−1^, *n* = 5) or vehicle (*n* = 5). I,J) The picture and weight statistics of tumors treated with alminoprofen (50 mg kg^−1^) or vehicle, *P* = 0.0022. K) The effect of alminoprofen on the levels of p‐STAT3, p‐JAK2 and C21orf58 proteins were examined by western blot in tumors treated with alminoprofen (50 mg kg^−1^) or vehicle. All **P< 0.01, ***P< 0.001, ns: not significant.

Figure S9D (Supporting Information) indicated the therapeutic potential of C21orf58 in HCC. We used virtual screening techniques to identify active compounds from the vast ZINC database, and the compounds could bind to C21orf58 and repress its malignant roles. Among the candidate compounds, alminoprofen, a nonsteroidal anti‐inflammatory drug in clinics but not reported in cancer therapy, stood out as a prime candidate for further experimental exploration due to its remarkable binding scores, plummeting to as low as −8.8 kcal mol^−1^. To detect the binding site where the active compound attached to the C21orf58 protein, it was revealed that the ligand of alminoprofen with C21orf58 established eight van der Waals bonds using the BIOVIA Discovery Studio Visualizer Tool, involving GLN213, THR210, GLN206, ALA193, PRO194, ARG139, ASP154, and ASN260. Additionally, conventional hydrogen bonds were established with ARG198 and SER190 (**Figure** **7**A). A unique interaction surfaced involving carbon hydrogen bonding between the alminoprofen and the amino acid residue PRO191 of C21orf58. Besides, interactions between them encompassed six alkyl bonds and pi‐alkyl bonds with VAL275, ALA209, LEU156, and ILE212 (Figure 7B,C). Alkyl and hydrogen bonds emerged as the most predominant modes of interactions. The results of H Nuclear Magnetic Resonance Spectra suggested that alminoprofen could bind on the C21orf58 protein (Figure S10A, Supporting Information). Then, the inhibition of alminoprofen on STAT3 activity and growth viability in HCC cells were evaluated. Cell viability and clone formation assays found that alminoprofen displayed an excellent inhibitory effect on cell growth of different malignant HCC cells (Figure 7D and Figure S10B, Supporting Information). Meanwhile, with the increase of concentration, alminoprofen significantly reduced the phosphorylation of STAT3 in HCC cells (Figure 7E), and similar results were detected by in vitro kinase activity and protein phosphorylation assays that alminoprofen completely blocked the activation of STAT3 mediated by C21orf58 (Figure 7F,G). Moreover, alminoprofen remarkably decreased the growth rate, volume and weight of Huh7‐derived xenografts when compared with vehicle (Figure 7H–J). A significant reduction of p‐STAT3 was also observed in alminoprofen‐treated tumors, accompanying by a decrease of p‐JAK2 (Figure 7K). Therefore, our results indicated an impressive suppression of alminoprofen on the tumorigenesis of HCC cells, suggesting that it was an alternative drug of HCC treatment.

## 3 Discussion

The biofunction of C21orf58 was still elusive.^[^ ^6^ ^]^ Its expression was abnormal in lung adenocarcinoma cell lines and breast cancer tissues, and inferred with the malignancy of breast cancer.^[^ ^7^ ^]^ However, the biological function and molecular mechanism of C21orf58 in cancer remain unclear. Our study demonstrated that the expression of C21orf58 was significantly increased in HCC tissues, which was consistent with the expression pattern in breast cancer.^[^ ^6b^ ^]^ C21orf58 expression positively correlated with clinical tumor size, negatively associated with HCC patients’ prognosis, which suggested that C21orf58 might be an oncogene in HCC. Our results demonstrated that C21orf58 exerted a role of malignant factor to promote HCC cell growth, clone formation and tumorigenesis of HCC cells, which was consistent with the clinical analysis.

Overactivation of STAT3 could promote tumor growth either directly through tumor autonomic mechanisms or indirectly by modulating antitumor responses.^[^ ^16^ ^]^ In our study, we observed that C21orf58 accelerated the cell cycle, and enhanced the expression of p‐STAT3 and its target genes (CyclinD1 and IL‐6), which were involved in promoting cell growth.^[^ ^8b^ ^]^ Besides, the expression of p‐STAT3 and C21orf58 showed a positive correlation in clinical HCC tissues. Moreover, inhibition of STAT3 and JAK2 by knockdown or using inhibitors could block the growth promoting phenotype mediated by C21orf58 in HCC cells. Therefore, we validated that C21orf58 modulated the cell growth by activating STAT3 signaling.

Non‐receptor tyrosine kinase JAK2 phosphorylated the STAT3 at Tyr705 to activate STAT3 activity, then causing STAT3 dimerization and translocation to the nucleus to induce transcription of target genes.^[^ ^17^ ^]^ Therefore, JAK2 was indispensable for STAT3 activation. Mechanistically, we found that C21orf58 simultaneously interacted with NTD domain of STAT3 and SH2 domain of JAK2, promoted the binding between JAK2 and STAT3, and formed a STAT3‐activating complex JAK2/C21orf58/STAT3, which evidently improved activity of JAK2 kinase and caused the activation of STAT3 cascades. Constitutively activated mutations of STAT3 could lead to STAT3 overactivation in cancers.^[^ ^10^, ^18^ ^]^ Fortunately, we confirmed that STAT3 constitutively activated mutations preferred to form ternary complex with JAK and C21orf58 and made them hyperactivation. Our results discovered a new regulator of JAK2/STAT3 signaling and provided a new explanation of abnormal activation of wildtype and constitutively mutated STAT3. Primarily, three regulatory mechanisms were reported to control the magnitude and duration of STAT3 signaling: 1) by removing phosphate group using the enzyme tyrosine phosphatases.^[^ ^19^ ^]^ 2) by adding a small ubiquitin‐like modifier by the protein inhibitor of activated STAT (PIAS) onto proteins.^[^ ^20^ ^]^ 3) by forming a protein complex through the interaction between suppressors of cytokine signaling (SOCS) with different proteins.^[^ ^21^ ^]^ Our study explored a new regulatory mechanism that through promoting the interaction by forming a ternary activating complex, and detailed the network between upstream factors and STAT3.

Many studies found that activation of STAT3 was a common cause of resistance to targeted therapies and chemotherapies in many cancers.^[^ ^22^ ^]^ STAT3 activation was implied in treatment failure of EGFR/ERK‐targeted and HER‐2‐targeted therapies in various cancers.^[^ ^23^ ^]^ It was also observed that the sorafenib‐resistant HCC cells highly expressed activated‐STAT3, and STAT3 overactivated HCC was more aggressive and drug‐resistant.^[^ ^10^, ^24^ ^]^ Our data showed that C21orf58 was highly expressed in sorafenib‐tolerated HCC cells and positively associated with sorafenib resistance. Besides, when C21orf58 expression was inhibited, drug‐resistant HCC cells were more sensitive to sorafenib and growth activity was significantly repressed. We explored a new reason to mediated sorafenib resistance and provided C21orf58 served as a potential therapeutic target of sorafenib insensitive HCC cells.

Forward feedback loop of STAT3 gave malignant capacities to the cancers. Through this feedback loop, STAT3 activated the transcription of target gene IL6, which was the initiator of IL6/JAK2/STAT3 and subsequently activated the STAT3 activity by JAK2 to amplify the effects of signaling.^[^ ^23b^ ^]^ In our study, the enhancement of IL‐6 and p‐JAK2 expression were detected in C21orf58‐overexpressed HCC cells accompanied by increase of p‐STAT3, which might be the results caused by forward feedback loop of STAT3, amplifying STAT3 signaling and endowing HCC with malignancies.

Due to the activation of STAT3 frequently occurred in many human cancers, and it was correlated with the malignant properties of cancers. Especially, aberrant STAT3 could promote HCC development and aggression. Therefore, inhibiting STAT3 signaling by compounds was one of the favorite strategies to develop new cancer treatments. However, STAT3 inhibitors did not show impressive anti‐tumors effects in clinical trials because of significant drawbacks, including multiple side effects. We used virtual screening technique to simulate the binding of C21orf58, STAT3 and JAK2 proteins to each other, to define the probable binding amino acid residues between them, and to screen potential small molecular compounds targeted to C21orf58. Due to its remarkable binding scores, alminoprofen, a nonsteroidal anti‐inflammatory drug of the phenylpropionic acid class, stood out as a prime candidate for further experimental exploration. Through the investigation of inhibitory effect on growth of HCC cells and phosphorylation of STAT3, we found that alminoprofen, never being studied in cancer treatment, had an effective anti‐tumor role on HCC cells through binding on C21orf58 and destroying the formation of C21orf58/JAK2/STAT3 complex, resulting in diminishment of STAT3 activity. We revealed that targeting C21orf58 was an attractive way of HCC treatment, and alminoprofen had a promising potential on HCC therapy.

Based on our results, there are several research aspects that can be further explored: 1) Subcellular localization results show that the C21orf58 protein is localized in the cytoplasm and nucleus. Our study just explored the role of C21orf58 in the cytoplasm; the features and potential mechanism of the C21orf58 in the nucleus are valuable to be deeply investigated. 2) The activation of STAT3 is commonly associated with inflammation and immunity. This study only showed that C21orf58 overactivated STAT3 cascades to regulate cell growth and drug resistance. Our future plan is to explore the vital role of C21orf58 in tumor immunity.

## 4 Conclusions

**Figure 8:**
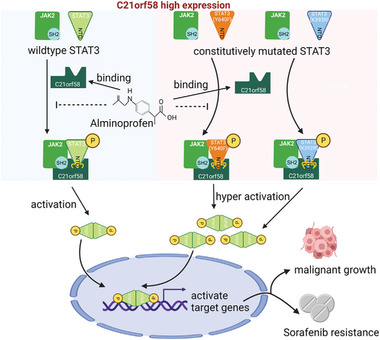
Schematic representation of the molecular mechanism that C21orf58 played oncogenic adaptor role on promoting cell growth and sorafenib resistance by activating STAT3 cascades in HCC cells with wild‐type STAT3 or constitutively mutated STAT3.

We demonstrated that C21orf58 displayed oncogenic role in promoting cell growth, tumorigenesis and sorafenib resistance of HCC cells by abnormal activation of STAT3 signaling. Mechanistically, we revealed that C21orf58 formed a ternary complex with JAK2 and STAT3, prompted JAK2 to phosphorylate wildtype and constitutively activated STAT3, leading to overactivation of STAT3 cascades and poor prognosis of patients. Besides, we identified that a clinical anti‐inflammatory drug alminoprofen targeted C21orf58, displayed an attractive anti‐tumor efficacy on HCC treatment by preventing the formation of JAK2/C21orf58/STAT3 complex, resulting in suppression of STAT3 cascades (**Figure** **8**). Our study validated the malignant adaptor role of C21orf58 and discovered a novel regulatory manner of JAK2/STAT3 signaling, and revealed the therapeutic potential of targeting C21orf58 in HCC.

## 5 Experimental Section

### Cell Lines and Tumor Samples

HepG2, Hep3B, Huh‐7 cell lines were purchased from Cell Bank of Type Culture Collection of Chinese Academy of Sciences, Shanghai Institute of Cell Biology, Chinese Academy of Sciences (CAS). MHCC97‐H and HEK293T cells were obtained from Xie's lab, Shanghai Institute of Nutrition and Health, CAS. Dulbecco's modified Eagle's medium (DMEM, Hyclone) supplemented with 1% Penicillin‐Streptomycin Solution (GP3108, Genview) and 10% fetal bovine serum (FBS, A0500‐3010, Cegrogen Biotech) were used to culture the cells, cell lines were incubated at 37 °C in a humidified incubator containing 5% CO_2_/95% air (v/v). All experiments performed in studies involving human participants or animals were approved by the Ethical Committee of School of Life Sciences, Central South University (Changsha, China; agreement no. 2021‐2‐22 and 2021‐1‐16). All patients gave their written consent before inclusion in the study. All the mouse experimental procedures were performed according to the protocols approved by the Institutional Animal Care and Use Committee of Central South University.

### Reagent and Antibodies

Anti‐C21orf58 (GeneTex, GTX46510), anti‐EGF receptor (#4267), anti‐phospho‐EGF receptor (Tyr1068) (#3777), anti‐ERK1/2 (#9102), anti‐phospho‐ ERK1/2 (Thr202/Tyr204) (#4370), anti‐STAT3 (#4904), anti‐phospho‐STAT3 (Tyr705) (#9145), anti‐AKT (pan) (#4691), anti‐Ki‐67(8D5) (#9449), anti‐phospho‐AKT (Ser473) (#4060), anti‐JAK2 (#3230), anti‐HA‐Tag (#3724) and anti‐phospho‐Jak2(Tyr1007/1008) (#3776) were obtained from Cell Signaling Technology. Anti‐STAT3 (sc‐8019) was obtained from Santa Cruz Biotechnology. Anti‐GST mouse monoclonal Antibody was purchased from TransGen Biotech (HT601‐01). Anti‐Flag M2 (F1804), goat anti‐Mouse IgG Antibody, peroxidase conjugated, H+L (AP124P) and goat anti‐rabbit IgG antibody, peroxidase conjugated (AP132P) were obtained from Sigma. Anti‐tubulin (TA503129) was purchased from OriGene. Anti‐GAPDH (AP0066) was obtained from Bioworld Technology. Goat anti‐rabbit IgG H&L (Alexa Fluor 488) (ab150077) and goat anti‐mouse IgG (H+L) highly cross‐adsorbed secondary antibody, alexa fluor 568 (A11031) were obtained from Abcam. Recombinant human STAT3 GST (N‐Term) protein was purchased from Novus Biologicals (H00006774‐P01). Protein A/G plus‐agarose (sc‐2003) was purchased from Santa Cruz Biotechnology. Anti‐Flag immunomagnetic beads (B26101) and anti‐HA immunomagnetic beads (B26201) were purchased from Bimake. Fedratinib (TG101348) (S2736) and Stattic (S7024) were obtained from Selleck.

### Plasmid Construction and Transfection

Flag‐C21orf58, HA‐STAT3 truncations (M1∼5 and N5), HA‐STAT3(K392R and Y640F) and shRNA plasmids were constructed using lentiviral construct. HA‐STAT3 (P34147), HA‐JAK2 (P35961) and pCMV‐MCS‐3×HA‐Neo (P13306) were purchased from the Miaoling Plasmid Sharing Platform, Wuhan, China. Stable‐expressing cells were sorted by flow cytometry. Green fluorescent protein (GFP) positive cells were screened. Then, obtained cells were cultured and expanded, and western blot was used to identify the overexpression of C21orf58. The sequences of primers and shRNAs were shown in Tables S1 and S2 (Supporting Information).

### RNA Extraction and Quantitative PCR

Total RNA was isolated from cells using RNA simple Total RNA Kit (DP419, TIANGEN). cDNA was synthesized from 1 µg of total RNA using the FastKing gDNA Dispelling RT SuperMix Kit (KR118, TIANGEN) as described by the manufacture. Realtime quantitative PCR (qRT‐PCR) reactions were performed using qRT‐PCR Kit (11201ES03, YEASEN). Sequence of qRT‐PCR primers were list in Table S3 (Supporting Information). The data were obtained using the method as previously described.^[^ ^25^ ^]^

### Western Blot

Protein lysates were extracted using RIPA (0.1% SDS, 1% NP‐40, 0.5% sodium deoxycholate, 50 × 10^−3^ m Tris (pH = 7.4)) buffer supplemented with phosphatase and protease inhibitors (Sigma). The expression levels of proteins were detected by western blot following previous method.^[^ ^26^ ^]^

### Cell Growth Assay

MTT (3‐(4,5‐dimethyl‐2‐thiazolyl)−2,5‐diphenyl‐2‐H‐tetrazolium bromide), CCK‐8 (Cell Counting Kit‐8) and crystal violet assays were performed to examine the effect of C21orf58 on growth of HCC cells. In MTT and CCK‐8 assays, each well of 96‐well plate was seeded with 2 or 3 × 10^3^ cells. 20 µL 3‐(4, 5‐dimethylthiazol‐2‐yl)−2, 5‐diphenylt etrazolium bromide solution or CCK‐8 solution was added to each well and incubated for 6 h at 37 °C. Then medium of plate examined by MTT was discarded, the formazan produced was dissolved in 200 µL dimethyl sulfoxide (DMSO). Cell viability was examined at the absorbance of 490 nm (MTT) or 450 nm (CCK8). In crystal violet assay, equal number of cells and control cells were seeded in six‐well plates and cultured for a week, medium was refreshed every two days. After 7 d, medium was discarded and added 0.5% crystal violet solution in 20% methanol. 10 min later, the fixed cells were washed with ddH_2_O and taken pictures.

### Clone Formation Assay

Clone formation was assessed by soft agar assay, 2000 cells per well were plated in 24‐well, flat‐bottomed plates using a two‐layer soft agar system in a volume of 400 µL per well. After 12 d of incubation, colonies were counted and measured.

### Cell Cycle Assay

Cells were harvested, washed twice with PBS buffer and fixed with 70% ethanol (pre‐cooled) for 6 h at 4 °C. Fixed cells were washed, pelleted, re‐suspended in 600 µL PBS containing 60 µg RNase A and 6 µg propidium iodide (PI) in the dark at 37 °C for 30 min. Finally, cell cycle distributions of HCC cells were assessed by a FACSCalibur cytometer (BD Biosciences).

### In Vivo Tumorigenesis

The nude mice were maintained in specific pathogen‐free environments. Cells were injected subcutaneously into the 5 weeks old male nude mice. The tumors were randomly divided into two groups. siRNA processing condition: tumors were treated with siC21orf58 (5 nmol) or negative control siRNA twice a week. Durg processing condition: tumors were treated with alminoprofen (50 mg kg^−1^) or vehicle every day. Tumor volumes were measured every 2 d using calipers, and tumor volumes were calculated using the formula length × (width)^2^/2. At the end of the study, we surgically removed the tumors, weighed and processed. All animal experiments were performed in accordance with China FDA guidelines. Protocols were reviewed and approved by the Department of Laboratory Animals, Central South University.

### Immunohistochemistry Staining (IHC)

The tissue was fixed in ice‐cold acetone for 30 min, washed in 0.01 m phosphate‐buffered saline (PBS) for 3 × 5 min, blocked for 1 h in 0.01 m PBS supplemented with 0.3% Triton X‐100 and 5% normal goat serum, and then incubated with C21orf58, Ki‐67 antibody at 4 °C overnight. Subsequent procedures of IHC and analysis of proteins intensity followed previous approach.^[^ ^27^ ^]^

### Co‐Immunoprecipitation (Co‐IP) Assay

Cells were cotransfected with the indicated plasmids for 48 h and then lysed. Cell homogenates were incubated with constant agitation and then centrifuged. For each IP sample, the supernatant of the lysate was incubated overnight at 4 °C with 30 µL of Protein A/G PLUS‐Agarose beads (sc‐2003) and 3 µL of the indicated primary antibody on a rocking platform. Finally, the beads were washed 5–6 times with cold IP buffer and 1 × loading buffer was added and boiled.

### Immunofluorescence (IF) Assay

After washing with pre‐cold PBS, cells seed on coverslips were fixed in 4% pre‐cold paraformaldehyde for 15 min, blocked with 1% TritonX‐100 in 5% bovine serum albumin (BSA) for 1.5 h, and followed by incubation with primary antibody C21orf58 (GeneTex, GTX46510) and STAT3 (Santa Cruz, sc‐8019) overnight at 4 °C. Goat Anti‐Rabbit IgG H&L (Alexa Fluor 488) (ab150077) and Goat anti‐Mouse IgG (H+L) highly cross‐adsorbed secondary antibody, Alexa fluor 568 (A11031) were incubated with cells for 1 h. The nuclei were stained with DAPI (4′,6‐diamidino‐2‐phenylindole) dye and fluorescence was observed via fluorescence microscope (Nikon).

### Protein Purification

Proteins were purified using anti‐Flag/anti‐HA immune magnetic beads (Bimake, B26101, B26201) according to the manufactures.

### GST‐Pull Down

Take purified Flag‐C21orf58 (full length), HA‐JAK2, and GST‐STAT3 recombinant proteins and add respectively 5 × SDS protein loading buffer was denatured at 98 °C for 10 min to obtain an Input sample. Similarly, mix the purified Flag‐C21orf58, HA‐JAK2, and GST‐STAT3 recombinant proteins evenly to obtain a mixture. Incubate at 4 °C overnight, take a portion of the mixture, and add 5 × SDS protein loading buffer was denatured at 98 °C for 10 min to obtain Mix input samples. Take partially purified Flag‐C21orf58, HA‐JAK2, GST‐STAT3, and their mixture, add IP lysis buffer, and then add Anti Flag immune magnetic beads. Incubate overnight at 4 °C, clean with PBST the next day, then use a magnetic frame for magnetic separation to remove the supernatant, and finally 1 × SDS protein loading buffer was added to the precipitate, denatured at 98 °C for 10 min, and then separated by magnetic separation on a magnetic rack to obtain an IP sample. Input samples and IP samples are used for subsequent western blot experimental detection.

### Kinase Assay

Kinase‐Lumi Luminescent Kinase Assay Kit (S0150S, Beyotime) was used following its instruction. According to different experimental groups, add an appropriate amount of reaction buffer (PBS), kinase substrate (GST‐STAT3), ATP and kinase, and other reagents to the kinase reaction system. After incubating at room temperature for 40–60 min, transfer a portion of the reaction solution to a black 96‐well plate and add Kinase‐Lumi Chemiluminescence kinase detection reagent, mix well and react at room temperature for 10 min, then perform chemiluminescence detection using a multifunctional enzyme‐linked immunosorbent assay. Take another portion of the reaction solution and add 5 × SDS protein loading buffer, then incubate at 98 °C for 10 min. Subsequently, the levels of proteins were detected by western blot.

### Protein Receptors Preparation

I‐TASSER server was used for homology modeling to obtain the crystal structure of C21orf58 protein. The server employs an automated hierarchical protocol for predicting and annotating protein structures. The structure, with added hydrogen atoms, underwent energy minimization using the MMFF94x force field in MOE software for geometry optimization.^[^ ^28^ ^]^

### Preparation of Ligands

Ligands from the ZINC database (5000 compounds) were transformed from SDF to mol2 format using Open Babel.^[^ ^29^ ^]^ The process involved merging hydrogen atoms, assigning charges, and standardizing internal degrees of freedom. Ligand molecules were converted to dockable pdbqt format through Autodock tools for docking with Auto Dock 4.2 software, where fixed components were defined automatically, and all rotatable bonds were set as active for potential interactions.^[^ ^30^ ^]^

### Virtual Screening

The protein underwent virtual screening using MOE's High Throughput Conformational Search, involving molecular fragmentation, conformation exploration, and alignment of overlapping atoms. This process, detailed on www.chemcomp.com/MOE‐Molecular_Operating_Environment.htm/, quickly generated diverse conformations for virtual screening. Compounds were selected based on features indicating effective interaction with the protein. From the ZINC database, compounds with a molecular weight below 500 kDa and RMSD value ≤1 were isolated. The search, accessible at https://zinc15.docking.org/substances/home/, aimed at identifying innovative molecules with distinctive structural attributes. A stringent filtering process, guided by Lipinski's rule of five, identified drug‐like compounds suitable for binding with the C21orf58 protein, which were kept for further validation.^[^ ^31^ ^]^

### Molecular Docking

Molecular docking, a common method for studying protein‐ligand interactions, was used in this study. The target protein was identified through homology modeling with the I‐TASSER server. Docking sites were determined using PyRx software, removing water molecules not directly interacting with inhibitors. A specific protein chain was chosen for analysis.^[^ ^32^ ^]^ Autodock Vina was used to examine ligand‐protein interactions, with protein energy minimized before docking. Docking configurations were selected based on the most negative values of binding energy for further refinement.^[^ ^33^ ^]^

### Visualization

The intricate hydrogen bond interactions in the 2‐D complex structure of the receptor‐ligand were meticulously examined using Discovery Studio 4.5 software. This analysis aimed to identify specific interactions between individual amino acids in the receptor and corresponding ligands. Discovery Studio 4.5 visually represented hydrophobic and hydrogen bonds, along with their associated bond lengths, for each docking configuration.^[^ ^34^ ^]^

### Molecular Dynamic Simulation

Crystal structures of C21orf58, STAT3, and JAK2 proteins were obtained from the Protein Data Bank (PDB) and modeled, incorporating missing residues and eliminating non‐protein entities. The structures underwent solvation in a TIP3P water box, with added counterions for system neutrality. Using the Desmond module within Schrödinger Release 2020‐3, protein–protein interactions, binding interactions, and affinities were explored through 100 ns simulations using the OPLS 2005 force field at pH 7.4. The simulations maintained constant temperature and pressure, and hydrogen bond analysis was conducted for C21orf58‐STAT3, C21orf58‐JAK2, and JAK2‐STAT3 interactions. Stability assessments included RMSD and RMSF, while trajectory frames revealed atom‐level protein interactions. Radius of gyration analysis showcased structural changes during the 100 ns simulation, providing insights into compression dynamics.^[^ ^35^ ^]^

### Protein Purification by Nickel Column Affinity Chromatography

The human C21orf58 amino acid was cloned into a pET‐21a plasmid, then transformed and expressed in Rosetta (DE3). A total of 0.5 × 10^−3^ m isopropyl β‐D‐thiogalactoside (IPTG) was added to LB medium when OD_600_ = 0.6. *Escherichia coli* bacteria were lysed by sonication and centrifuged for 30 min at 4 °C, 12 000×*g*. The recombinant proteins were purified after incubating the supernatant with Ni‐NTA (QIAGEN) for 2 h at room temperature.

### H‐NMR

First, lyophilize the purified C21orf58 protein into powder, then dissolve the alminoprofen and C21orf58 protein powder in deuterated heavy water, and transfer them to two nuclear magnetic tubes. Next, use the superconducting Fourier transform nuclear magnetic resolution spectrometer (AVANCE III HD 600, Bruker, Germany) to perform 30 machine tests. Then, mix the two solutions evenly and incubate at room temperature for 30 min before further testing 30 times.

### Statistical Analysis

The correlations between the clinicopathological features and C21orf58 staining scores were analyzed using the chi‐square (*χ*2) test. Survival curves were plotted by the Kaplan‐Meier method and analyzed by the log‐rank test. Statistical analyses were performed by GraphPad Prism 8 software. R 4.2.2 was used to pre‐process the gene expression data obtained by RNA‐seq, and screened the genes with less than half of the null expression value in the group. The results are representative of at least three independent experiments performed in triplicate and are expressed as the means ±SD. The data were analyzed using Student's t test. When the statistical result is *P* < 0.05, the results are significantly different, and when *P* < 0.01, the results are extremely different.

## Conflict of Interest

The authors declare no conflict of interest.

## Author Contributions

H.J. and Y.W. contributed equally to this work. Conceptualization: H.J. and S.Z.; Performing experiments, data analysis: H.J., Y.W., R.Y., K.L.,D.W., S.S.E., X.D., F.L.; Supervision, project administration, funding acquisition: H.J., X.D., S.Z.; Manuscript writing: H.J., Y.W.; Writing—review, editing: L.H., J.L., X.D.. All authors have read, agreed to the published version of the manuscript.

## Supporting information

Supporting Information

## Data Availability

The data that support the findings of this study are available from the corresponding author upon reasonable request.
