# Supplementary material for: Targeting C21orf58 is a Novel Treatment Strategy of Hepatocellular Carcinoma by Disrupting the Formation of JAK2/C21orf58/STAT3 Complex
Source: Adv Sci (Weinh). 2024 Feb 11;11(15):2306623. doi: 10.1002/advs.202306623 (PMC11022693; doi:10.1002/advs.202306623)
Supplement: Supplementary file 1 — Supporting Information [file ADVS-11-2306623-s001.pdf]

ADVANCED SCIENCE
Open Access

Supporting Information

for *Adv. Sci.*, DOI 10.1002/advs.202306623

Targeting C21orf58 is a Novel Treatment Strategy of Hepatocellular Carcinoma by Disrupting the Formation of JAK2/C21orf58/STAT3 Complex

Hao Jiang, Yang Wang, Doudou Wen, Rongji Yu, Sayed S Esa, Kefeng Lv, Qing Feng, Jing Liu, Faxiang Li, Lan He, Xiaotang Di* and Shubing Zhang**

Supplementary figure legend, figures and tables

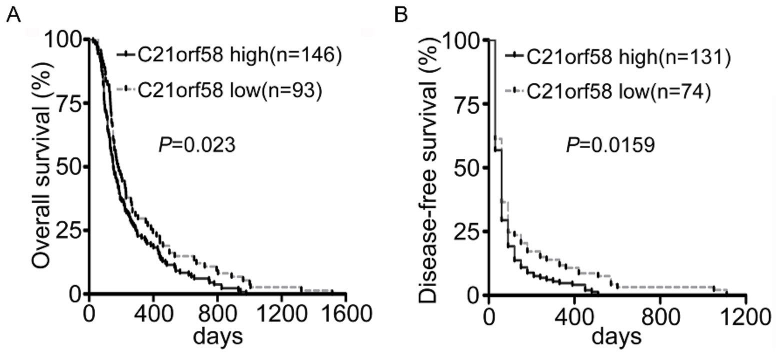

Fig. S1. The overall survival and disease-free survival of HCC patients with high and low C21orf58 expression. (A) Correlation analysis of C21orf58 expression with overall survival time (OS, P=0.023) in HCC patients. (B) Correlation analysis of C21orf58 expression with disease-free survival time (DSF, P=0.0159) in HCC patients.

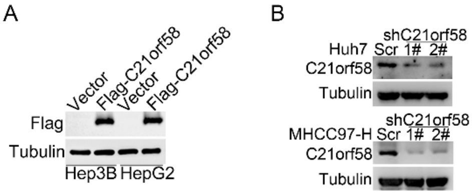

Fig. S2. The stable overexpression and knockdown of C21orf58 HCC cell lines were constructed. (A) The overexpression of C21orf58 in HepG2 and Hep3B cells were examined by western blot. (B) The knockdown of C21orf58 expression in Huh7 and MHCC97-H cells were examined by western blot. Scr: Scramble.

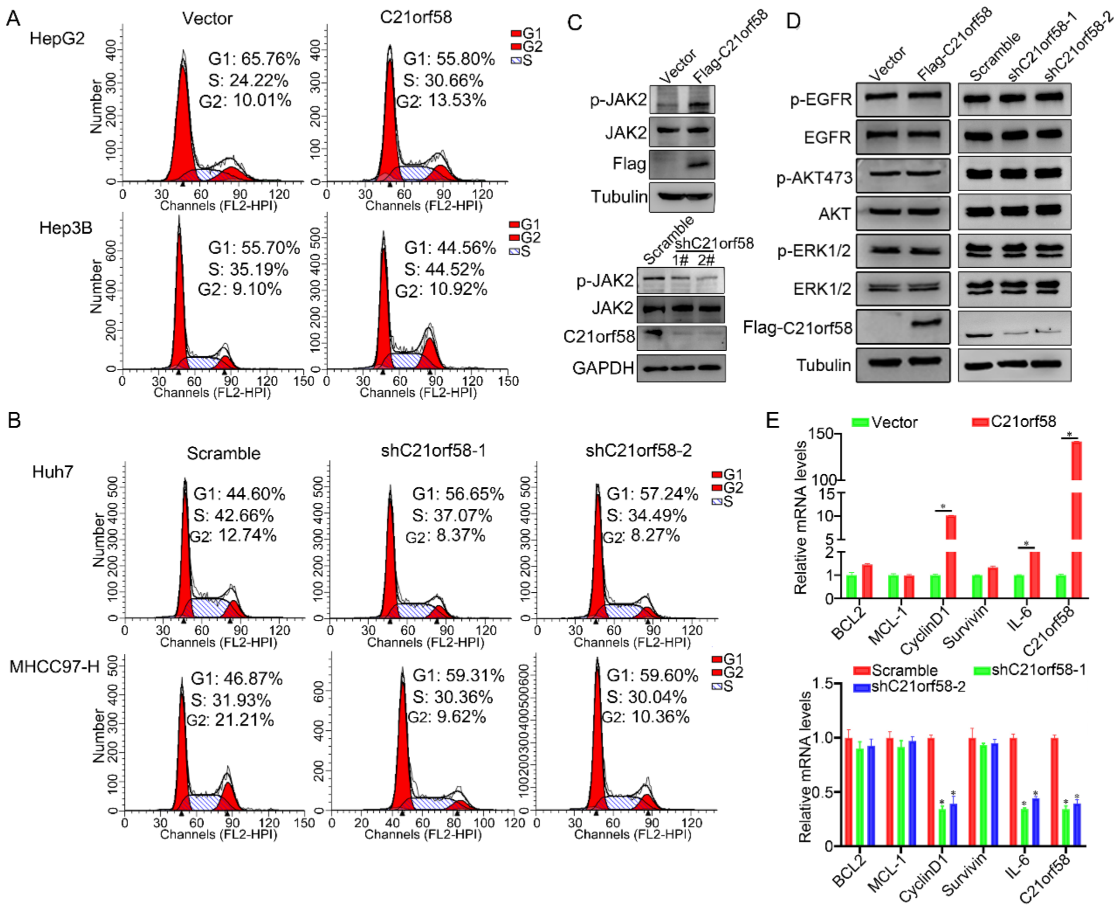

Fig. S3. C21orf58 affected the distribution of cell cycle in stably HCC cell lines. (A), (B) The distribution of cell cycle in C21orf58 overexpressing and knocking down HCC cells and their control cells. (C) C21orf58 enhanced the expression of p-JAK2, but did not alter the expression of JAK2 (D). C21orf58 had no effect on EGFR signal pathway. (E) The effect of C21orf58 on downstream genes of STAT3 signaling.

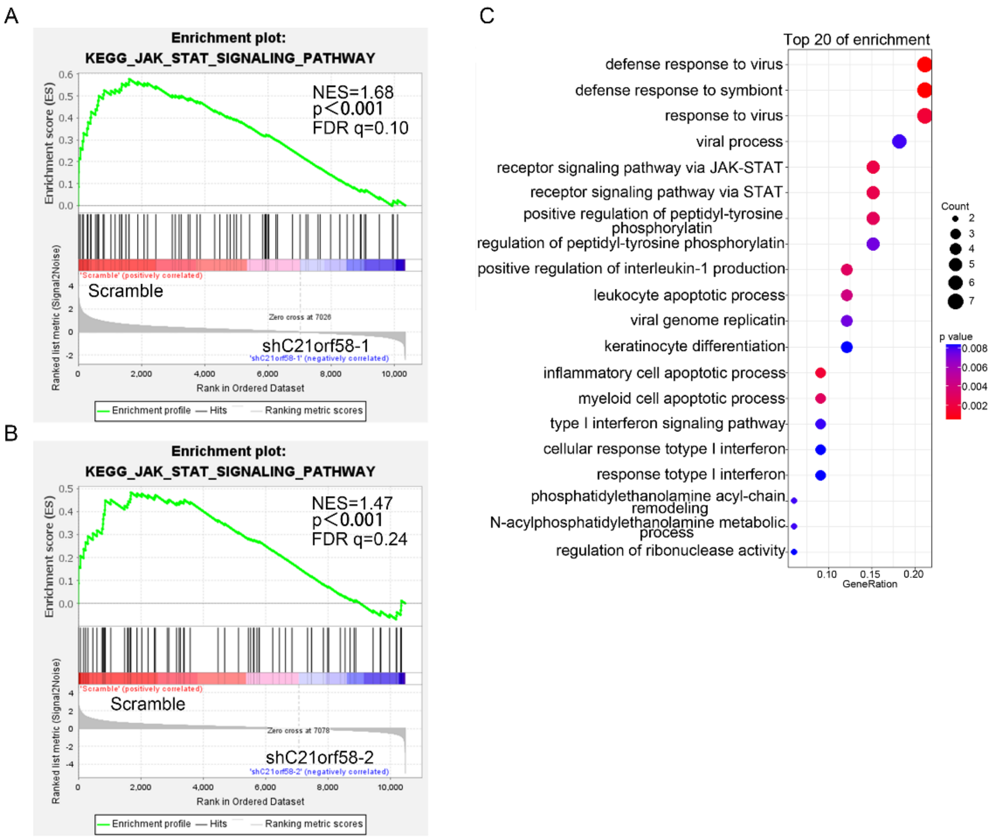

Fig. S4. C21orf58 activated the JAK-STAT signaling pathway. (A), (B) The gene set enrichment analysis (GSEA) plot of JAK-STAT signaling pathway based on the RNA-seq data from control and C21orf58 knockdown HCC cells (shC21orf58-1 and shC21orf58-2, n=3 per group). NES, normalized enrichment score. (C) The top 20 enrichment of differentially expressed genes by GO assay.

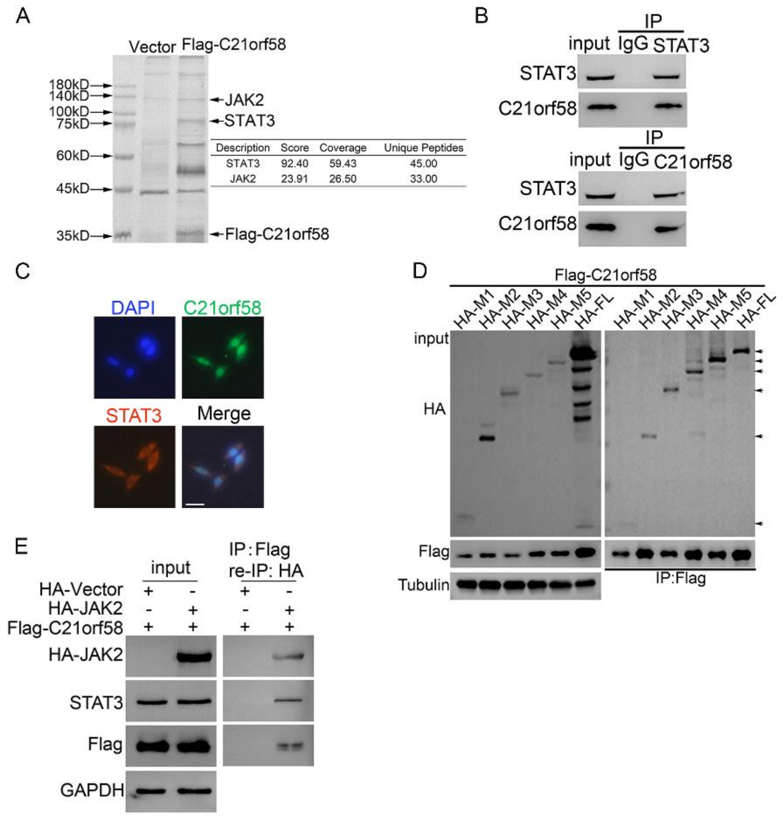

Fig. S5. C21orf58 simultaneously interacted with JAK2 and STAT3 to form a ternary complex. (A) Co-immunoprecipitation combined with mass spectrometry assay indicated that STAT3 and JAK2 proteins were the candidate proteins interacted with C21orf58. (B) The endogenous interaction between C21orf58 and STAT3 in HepG2 cells. (C) Localization of C21orf58 and STAT3 protein in HepG2 cells was examined by immunofluorescence assay, scale bar=25μm. (D) The interaction between C21orf58 and truncations of STAT3 was examined by immunoprecipitation in HEK293T cells. (E) immunoprecipitation-re-immunoprecipitation (IP-re-IP) assay was performed to conform that C21orf58 formed a ternary complex with JAK2 and STAT3 in HCC cells by direct interaction.

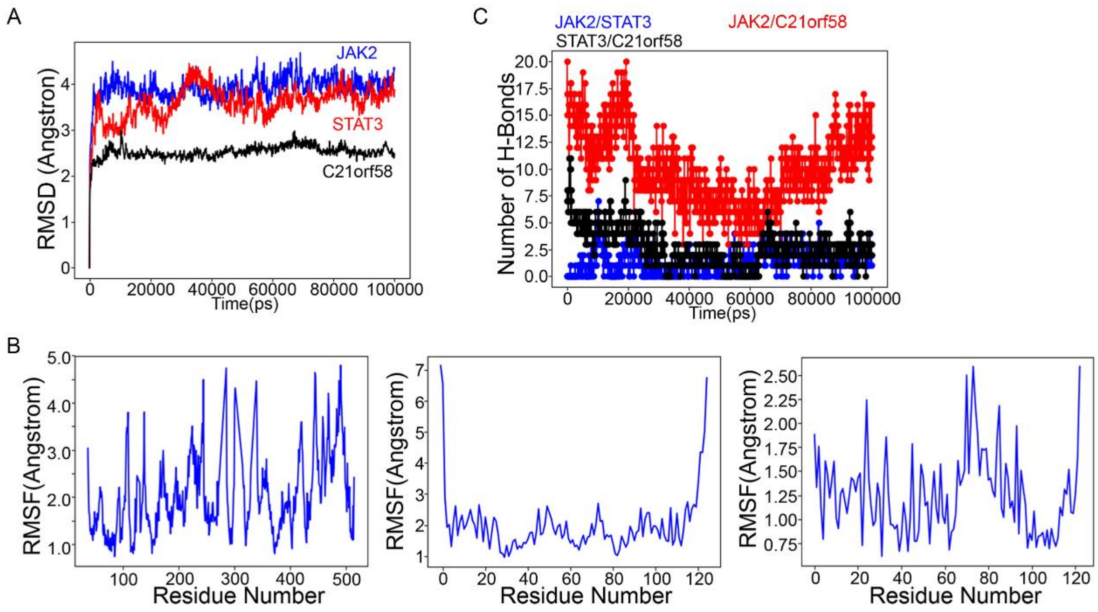

Fig. S6. Molecular dynamics simulation within C21orf58, STAT3, and JAK2. (A) C21orf58, STAT3, and JAK2 protein RMSD plots. (B) hydrogen bonds within C21orf58, STAT3, and JAK2 complex interactions. (C) C21orf58, STAT3, and JAK2 protein fluctuations in the RMSF plot.

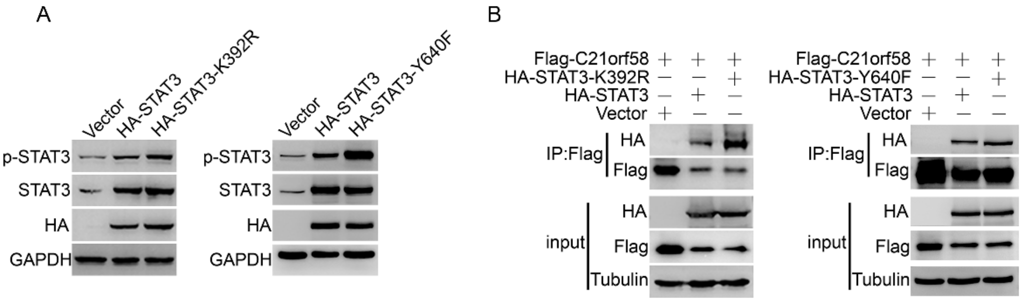

Fig. S7. C21orf58 preferred to interact with constitutively mutated STAT3. (A) Constitutively activated mutations K392R and Y640F significantly enhanced the activity of STAT3. (B) constitutively mutated STAT3 (K392R and Y640F) preferred to interact with C21orf58 when compared with wildtype STAT3.

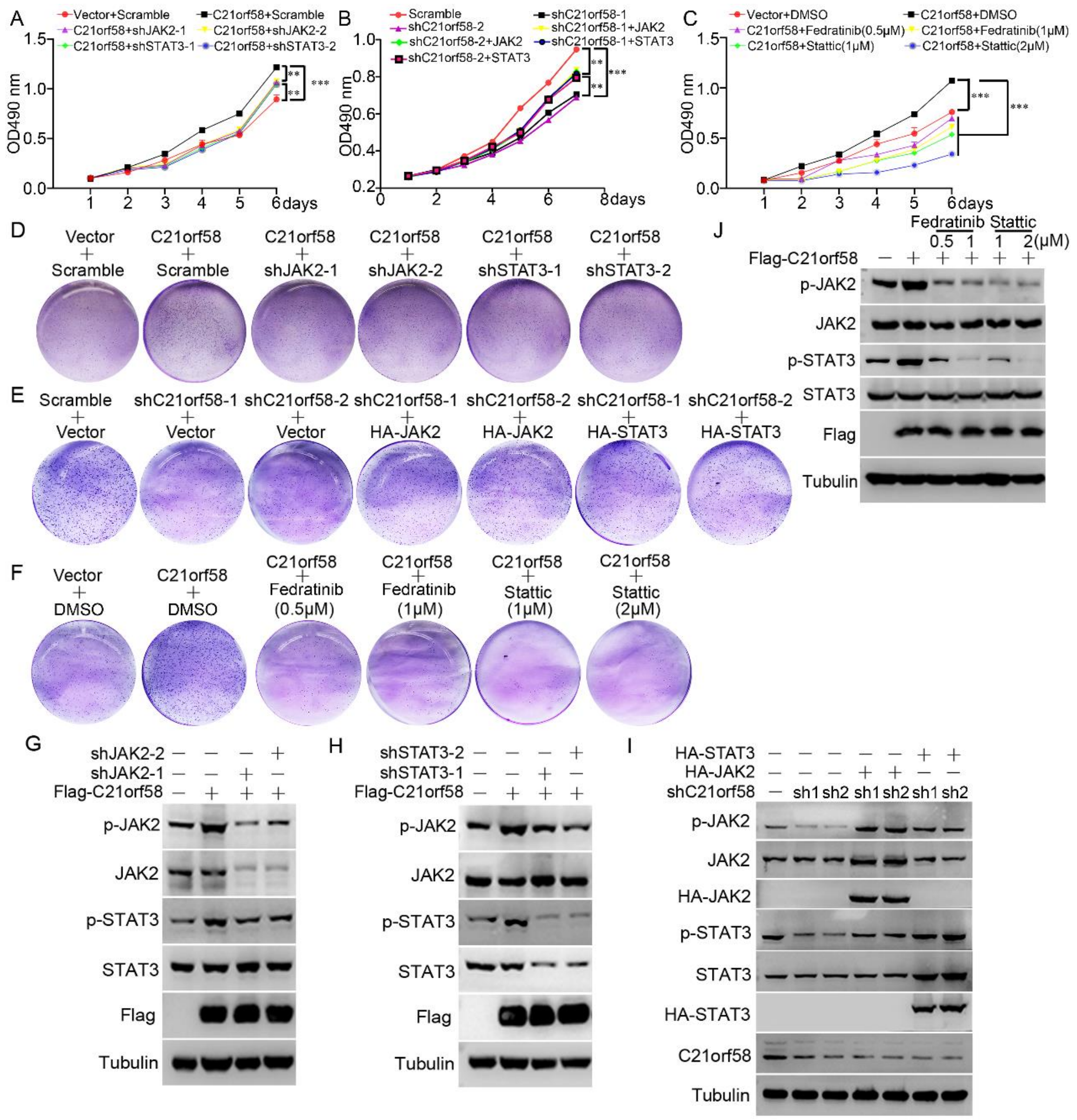

Fig. S8. Inhibition of JAK2/STAT3 signaling rescued the growth-promoting phenotype mediated by C21orf58. (A), (D) Attenuated JAK2 or STAT3 expression inhibited the growth ability and clone formation of HCC cells overexpressing C21orf58. (B), (E) Forced expression of JAK2 or STAT3 elevated the growth ability and clone formation of C21orf58 knockdown HCC cells. (C), (F) Inhibition of JAK2 or STAT3 activity with their inhibitors (JAK2 inhibitor Fedratinib and STAT3 inhibitor Stattic) suppressed the growth of C21orf58-overexpressed HCC cells. (G), (H) Reduced JAK2 or STAT3 expression diminished the activity of STAT3 in HCC cells with C21orf58 overexpression. (I) Upregulated JAK2 or STAT3 expression normalized the

activity of STAT3 in C21orf58 knockdown HCC cells. (J) JAK2 or STAT3 inhibitor dramatically decreased the level of p-STAT3(Y705) in C21orf58- overexpressed HCC cells. All ** $P<0.01$, *** $P<0.001$.

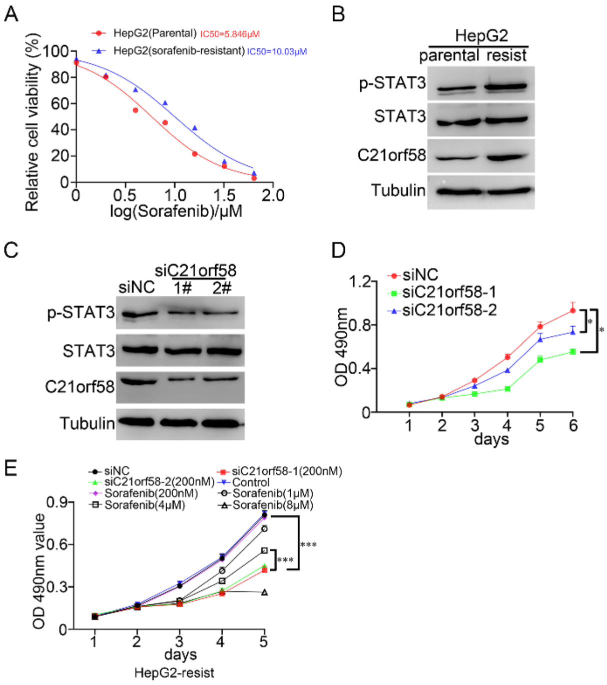

Fig. S9. Construction of sorafenib-resistant HCC cells and reduction of C21orf58 using siRNA could repress the malignant growth of HCC cells. (A) Construction of sorafenib-resistant HepG2 cells, which were not vulnerable to sorafenib compared with their parental cells. IC50 was the 50% inhibiting concentration. (B) The expression of C21orf58 and p-STAT3(Y705) were increased in sorafenib-resistant HepG2 cells. (C) Western blot examined the inhibition efficiency of siRNA on C21orf58 protein expression. (D) The viability of HepG2 cells treated with siC21orf58 and negative control siRNA (siNC) for 6 days. (E) Inhibition of C21orf58 expression using siRNA was effectively to repress the cell growth of HCC cells with sorafenib resistance. All * *P*<0.05.

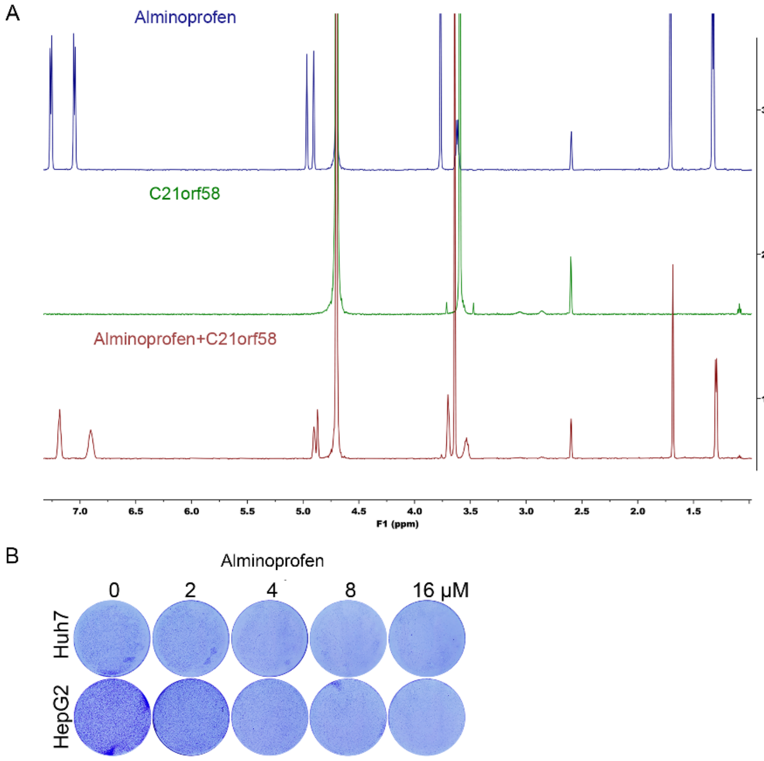

Fig. S10. Alminoprofen could bind on the C21orf58 protein and inhibit the colony formation of HCC cells. (A) H Nuclear Magnetic Resonance Spectra of alminoprofen, C21orf58 protein, alminoprofen+C21orf58 protein. (B) The inhibitory effect of alminoprofen on the colony formation of 993 HepG2 and Huh7 cells.

Table S1 Primers of overexpression plasmids

Genes	Sequence (5' to 3')
Flag-C21orf58	F: CCCTCGAGATGGCCATCCTGCCCGGAGA<br>R: CGGGATCCCGGGTGGGCCAGGCGTCCACA
HA-STAT3 (K392R)	F: TTAACATTCTGGGCACAAACACAAGAGTGATGAAC<br>R: GTTGGATTCTTCCATGTTCATCACTCTTGTGTTTG
HA-STAT3 (Y640F)	F: AGATCCAGTCCGTGGAACCATTCACAAAGCAGCAG<br>R: CATGTTGTTCAGCTGCTGCTTTGTGAATGGTTCCA
HA-STAT3-N5	F: CCAAGCTTATGCACCCCACAGCAGCCGTGGTGA<br>R: CCCTCGAGCATGGGGGAGGTAGCGCACTCC
HA-STAT3-M1	F: CCAAGCTTATGGCCCAATGGAATCAGCTAC<br>R: CCCTCGAGGTTGGCCTGGCCCCCTTGCTG
HA-STAT3-M2	F: CCAAGCTTATGGCCCAATGGAATCAGCTAC<br>R: CCCTCGAGGGCACTTTTCATTAAGTTTCTAAA
HA-STAT3-M3	F: CCAAGCTTATGGCCCAATGGAATCAGCTAC<br>R: CCCTCGAGGGAGATCACCACAACTGGCAAG
HA-STAT3-M4	F: CCAAGCTTATGGCCCAATGGAATCAGCTAC<br>R: CCCTCGAGGATGTACCCTTCGTTCCAAAGG
HA-STAT3-M5	F: CCAAGCTTATGGCCCAATGGAATCAGCTAC<br>R: CCCTCGAGCCGACAATACTTTCCGAATGCC
JAK2-J1	F: GGGTTTAAACATGGGAATGGCCTGCCTTACGATGA<br>R: TTGCGGCCGCGGTGCAGTTAATCTATAATATCCATCA
JAK2-J2	F: GGGTTTAAACATGGGAATGGCCTGCCTTACGATGA<br>R: TTGCGGCCGCGGTCGTACGCCTTTAAAAATCTTTGTA
JAK2-J3	F: GGGTTTAAACATGGGAATGGCCTGCCTTACGATGA<br>R: TTGCGGCCGCGGCTCCACACTCCCAAAATTACCCTTG
His-C21orf58	F: CGGAATTCATGGCCATCCTGCCCGGAGA<br>R: CCCTCGAGGGGTGGGCCAGGCGTCCACA

Table S2 Primers of knockdown plasmids

	Sequence (5' to 3')
Scramble	F:CCGGCCTAAGGTTAAGTCGCCCTCGCTCGAGCGAG GGCGACTTAACCTTAGG<br>R:AATTCCCTAAGGTTAAGTCGCCCTCGCTCGAGCGAG GGCGACTTAACCTTAGG
shC21orf58-1	F:CCGGTCAACGGTCAGGAAGTATTAACTCGAGTTAAT ACTTCCTGACCGTTGATTTTTG<br>R:AATTCAAAAATCAACGGTCAGGAAGTATTAACTCGA GTTAATACTTCCTGACCGTTGA
shC21orf58-2	F:CCGGCTGAAGAGAAGGAGGGACCTTCTCGAGAAGG TCCCTCCTTCTCTTCAGTTTTTG<br>R:AATTCAAAAACTGAAGAGAAGGAGGGACCTTCTCGA GAAGGTCCCTCCTTCTCTTCAG
shSTAT3-1	F:CCGGCACCATTCATTGATGCAGTTTCTCGAGAAACT GCATCAATGAATGGTGTTTTTG<br>R:AATTCAAAAACACCATTCATTGATGCAGTTTCTCGAG AAACTGCATCAATGAATGGTG
shSTAT3-2	F:CCGGGCTGACCAACAATCCCAAGAACTCGAGTTCTT GGGATTGTTGGTCAGCTTTTTG<br>R:AATTCAAAAAGCTGACCAACAATCCCAAGAACTCGA GTTCTTGGGATTGTTGGTCAGC
shJAK2-1	F:CCGGGCCATCATACGAGATCTTAACCTCGAGGTTAA GATCTCGTATGATGGCTTTTTG<br>R:AATTCAAAAAGCCATCATACGAGATCTTAACCTCGA GGTTAAGATCTCGTATGATGGC
shJAK2-2	F:CCGGGCTTTGTCTTTCGTGTCATTACTCGAGTAATGA CACGAAAGACAAAGCTTTTTG<br>R:AATTCAAAAAGCTTTGTCTTTCGTGTCATTACTCGAG TAATGACACGAAAGACAAAGC

Table S3 Primers of realtime-qPCR

Genes	Sequence (5' to 3')
C21orf58	F: AGACCCGCCAAGGATCATC<br>R: TTCCTGACCGTTGAGTAGGGA
BCL-2	F: AAAAATACAACATCACAGAGGAAGT<br>R: GTTTCCCCCTTGGCATGAGA
MCL-1	F: TGCTTCGGAAACTGGACATCA<br>R: TAGCCACAAAGGCACCAAAAG
CyclinD1	F: GCTGCGAAGTGGAAACCATC<br>R: CCTCCTTCTGCACACATTTGAA
Survivin	F: CACTGAGAACGAGCCAGACT<br>R: TTTCCTTTGCATGGGGTCGT
IL-6	F: ACTCACCTCTTCAGAACGAATTG<br>R: CCATCTTTGGAAGGTTCAGGTTG
GAPDH	F: ATGTTCGTCATGGGTGTGAA<br>R: CAGTGATGGCATGGACTGT

Table S4 Primers of siRNAs

Genes	Sequence (5' to 3')
Negative Control (NC)	F: UUCUCCGAACGUGUCACGUTT<br>R: ACGUGACACGUUCGGAGAATT
siC21orf58-1	F: GGCACAGACUCCCACUAAATT<br>R: UUUAGUGGGAGUCUGUGCCTT
siC21orf58-2	F: GGAGUAUUCCUUCAGCUAATT<br>R: UUAGCUGAAGGAAUACUCCTT
